# Massively Parallel Sequencing Reveals an Accumulation of *De Novo* Mutations and an Activating Mutation of *LPAR1* in a Patient with Metastatic Neuroblastoma

**DOI:** 10.1371/journal.pone.0077731

**Published:** 2013-10-16

**Authors:** Jun S. Wei, Peter Johansson, Li Chen, Young K. Song, Catherine Tolman, Samuel Li, Laura Hurd, Rajesh Patidar, Xinyu Wen, Thomas C. Badgett, Adam T. C. Cheuk, Jean-Claude Marshall, Patricia S. Steeg, José P. Vaqué Díez, Yanlin Yu, J. Silvio Gutkind, Javed Khan

**Affiliations:** 1 Oncogenomics Section, Pediatric Oncology Branch, Advanced Technology Center, National Cancer Institute, Bethesda, Maryland, United States of America; 2 The Advanced Biomedical Computing Center, SAIC-Frederick, Inc., National Cancer Institute, Frederick, Frederick, Maryland, United States of America; 3 Women’s Cancers Section, Laboratory of Molecular Pharmacology, Center for Cancer Research, National Cancer Institute, Bethesda, Maryland, United States of America; 4 Cell Growth Regulation Section, National Institute of Dental and Craniofacial Research, Bethesda, Maryland, United States of America; 5 Cancer Modeling Section, Laboratory of Cancer Biology and Genetics, Center for Cancer Research, National Cancer Institute, Bethesda, Maryland, United States of America; Tsan Yuk Hospital, Hospital Authority, China

## Abstract

Neuroblastoma is one of the most genomically heterogeneous childhood malignances studied to date, and the molecular events that occur during the course of the disease are not fully understood. Genomic studies in neuroblastoma have showed only a few recurrent mutations and a low somatic mutation burden. However, none of these studies has examined the mutations arising during the course of disease, nor have they systemically examined the expression of mutant genes. Here we performed genomic analyses on tumors taken during a 3.5 years disease course from a neuroblastoma patient (bone marrow biopsy at diagnosis, adrenal primary tumor taken at surgical resection, and a liver metastasis at autopsy). Whole genome sequencing of the index liver metastasis identified 44 non-synonymous somatic mutations in 42 genes (0.85 mutation/MB) and a large hemizygous deletion in the *ATRX* gene which has been recently reported in neuroblastoma. Of these 45 somatic alterations, 15 were also detected in the primary tumor and bone marrow biopsy, while the other 30 were unique to the index tumor, indicating accumulation of *de novo* mutations during therapy. Furthermore, transcriptome sequencing on the 3 tumors demonstrated only 3 out of the 15 commonly mutated genes (*LPAR1*, *GATA2*, and *NUFIP1*) had high level of expression of the mutant alleles, suggesting potential oncogenic driver roles of these mutated genes. Among them, the druggable G-protein coupled receptor *LPAR1* was highly expressed in all tumors. Cells expressing the LPAR1 R163W mutant demonstrated a significantly increased motility through elevated Rho signaling, but had no effect on growth. Therefore, this study highlights the need for multiple biopsies and sequencing during progression of a cancer and combinatorial DNA and RNA sequencing approach for systematic identification of expressed driver mutations.

## Introduction

Neuroblastoma is the most common extra-cranial solid tumor of childhood and has a poor prognosis for patients with metastatic disease [1]. Despite aggressive multimodal therapy, the current survival rate for high-risk neuroblastoma patients remains <40% [2]. Although usually seen in children under the age of 5 years (>95%), neuroblastoma occasionally occurs in adolescent and young adults [1]. In older patients this disease has a more indolent course, but relentlessly progresses with the eventual demise of patients resulting in an overall poorer prognosis than their younger counterparts [3-5]. Because of heterogeneity of neuroblastoma, the molecular basis of tumorigenesis during progression of this disease is not fully understood; and novel therapeutic targets are urgently needed to improve the survival rate in these patients.

Several recurring genomic alterations have been found often associated with poor outcome in neuroblastoma, which include *MYCN* amplification [6-8], 1p and 11q deletion [9], and *ALK* activating mutations [10-13]. Four recent large-scale studies of neuroblastoma using whole genome or exome sequencing revealed surprisingly few novel high-frequency recurrent somatic mutations, but reported on *ATRX* inactivating mutations preferentially found in adolescent and young adult patients [14], structural variants in genes *PTPRD, ODZ3, CSMD1*, and *ARID1A* [15,16], and mutations in *ARID1B*, *PTPN11*, *MYCN* and *NRAS* in neuroblastoma patients[16,17]. In these studies, it is uncertain whether somatically mutated alleles of these potential drivers are in fact expressed. Furthermore, although neuroblastoma tumors have been reported to harbor fewer somatic non-synonymous mutations (<1 non-synonymous mutation per megabase) [15,17] than those reported in adult cancers [18-20], it is still challenging to distinguish drivers from passengers, underlining the needs for other systematic approaches to pinpoint the casual events for tumor growth and metastasis. 

In order to identify all the non-synonymous somatic mutations present at the end of the course of a disease, we performed whole genome sequencing of genomic DNAs of an index liver metastasis and normal skin taken 3.5 years after initial diagnosis from a patient with neuroblastoma. Furthermore, we used ultra-deep sequencing to examine these somatic mutations in additional 5 tumor samples (a bone marrow metastasis at diagnosis and 4 different samples from the primary tumor removed by surgical resection) from the same patient. Finally, we performed transcriptome sequencing on the liver and bone marrow metastases and one of the primary tumors to identify potential driver oncogenes that have been present and expressed throughout the history of the tumor (Figure S1). These massively parallel sequencing experiments revealed an accumulation of *de novo* mutations and an activating mutation of *LPAR1* in this patient with metastatic neuroblastoma.

## Materials and Methods

### Ethics Statement

 This study was determined as “not human subjects research” by the Office of Human Subjects Research of National Institutes of Health under “Research involving coded Private Information or Biological Specimens”.

### Next-generation sequencing

 Whole genome sequencing was performed by Complete Genomics, Inc (CGI, Mountain View, CA) as described elsewhere [21]. CGI Analysis Pipeline 2.0 and CGAtools1.5.0 (http://cgatools.sourceforge.net/) was used in the sequence data analyses. Briefly, a total of 378 and 377 gigabases (Gbs) sequence was acquired for both tumor Met2 and normal skin DNA respectively from 35-base paired end reads of approximately 500-base genomic fragments using unchained combinatorial probe anchor ligation sequencing chemistry [21] (Table S1). Small variants (<50bp) including single nucleotide variant, small indels, and substitutions were called. Then somatic variants were identified by comparing tumor Met2 and skin DNA using CallDiff. We annotated small variants using ANNOVAR [22] for protein changing variants. In order to remove artifacts resulting from system errors of the platform, we used 119 normal human germ line DNAs including 69 from the Complete Genomics normal panel to reduce the false positive rate. Finally, we took all somatic protein changing mutations with somatic score ≥ -10 for further experimental validation using either Sanger or Ion Torrent semiconductor sequencing (Life Technology, Foster City, CA). Chromosomal copy number change for tumor genome was identified using average normalized against coverage in the normal skin with a 100kb window under the assumption of diploid in the normal skin sample. The summary of genomic changes in the tumor DNA is shown in a CIRCOS plot using CGAtools. 

 Transcriptome libraries were constructed from total RNA according to the manufacturer’s protocol for Total RNA-Seq Kits (Applied Biosystems, Foster City, CA). In brief, ribosomal RNA was first removed from total RNA using RiboMinus™ Eukaryote kits (Invitrogen, Carlsbad, CA), and then fragmented using RNase III. After ligated with adaptors and converted to cDNA using reverse transcription, the transcriptome libraries were size selected on 6% polyacrylamide TBE-Urea denaturing gels, and amplified by limited PCR cycles to avoid over-amplification. Transcriptome sequencing was performed using 50bp fragment library sequencing protocol on SOLiD™3 Plus or 4 systems (Applied Biosystems, Foster City, CA). We used Bioscope 1.2.1 (Applied Biosystems, Foster City, CA) to map the reads against human reference genome (build 36/hg18) in color space with the default setting. The genome coordinates of interest were then converted to build 37/hg19 using LiftOver batch coordinate conversion tools (http://genome.ucsc.edu/util.html). For each RNA sample, more than 141 million mapped reads (equivalent to approximate 6 Gb base pairs) were acquired. Sequence data used for this study are available at the NIH sequence read archive (SRA, http://www.ncbi.nlm.nih.gov/sra) under the accession number SRA048127.2. 

### Sequencing validation using Sanger and Ion Torrent semiconductor sequencing

 Sequencing validation was performed using either Sanger or Ion Torrent semiconductor sequencing (Applied Biosystems, Foster City, CA). In brief, genomic PCR primers were designed and synthesized across the mutations. Genomic PCR was performed to amplified DNA fragments of interest using an Access Array system (Fluidigm, South San Francisco, CA) according to the manufacturer’s instruction. PCR products were run on 2% agarose gels to ensure correct size before sequencing. Because the read length of semiconductor sequencer was <120 bp, amplicons of size >165bp were sequenced using Sanger method on an ABI 3700 capillary sequencer according to manufacturer’s instruction (Life Technology, Grand Island, NY). Semiconductor sequencing was performed on an Ion Torrent Personal Genome Machine using a 314 chip according to the Ampliseq protocol (Life Technology). All reads were aligned against human hg19 reference genome and mutations were called manually by visual inspection to ensure the positions and base pair changes of mutations. Due to the exquisite sensitivity of the semiconductor sequencing and possible chemistry errors, mutations having >5 variant reads and fraction of variant reads >1% were scored as true positive. Otherwise, they were excluded as contamination or sequencing artifacts. The primer sequences for validation of *ATRX* deletion are ATCTGGGTGCCCTACGTTTT and GTTACCCAGGCTGGAGTGC; *LPAR1* mutations, CTGTAGAGGGGTGCCATGTT and CAGGACCCAATACTCGGAGA.

### Site-directed mutagenesis and stably transfected cell lines

 To generate the corresponding R163W mutant LPAR1 expression construct, we used the QuickChange™ Site-Directed Mutagenesis Kit (Stratagene, La Jolla, CA). We then sub-cloned wild-type (WT) and mutant LPAR1 (MT) into the pCEFL2-SfiI vector with 3X HA tags. All expression constructs were sequence validated to ensure accuracy. To establish NIH3T3 cells stably expressing HA-LPAR1 constructs, cells were transfected with vector (control), WT, and MT constructs using Lipofectamine-Plus (Invitrogen, Carlsbad, CA), and then selected in 0.4 mg/ml of G418 (Sigma, St. Louis, MO). Comparable expression of WT and MT was ensured using Western blotting of LPAR1 using a rabbit polyclonal LPAR1 antibody (Novus Biologicals, Littleton, CO) and FACS by incubating cells with an anti-HA antibody (Covance, Princeton, NJ) (data not shown). An antibody against tubulin was purchased from Millipore (Damstadt, Germany). 

### Gene expression profiling of NIH3T3 cells

 To explore the signaling pathways of mutant LPAR1, we performed gene expression profiling for NIH3T3 cells expressing wild-type and mutant LPAR1 receptors using Affymetrix mouse 430 2.0 arrays according to the manufacturer’s protocol (Affymetrix, Santa Clara, CA). Serum was withdrawn from culture media for overnight before lysophosphatidic acid (LPA), the ligand of LPAR1, was added to the final concentration of 10 μM. Cells were harvested at 0, 1, and 2 hours for RNA extraction using a previously published protocol [23]. To estimate relative expression value, *X*, probe sets were summarized and normalized using the PLIER algorithm implemented in Affymetrix Power Tools v1.14.3.1 with option ‘plier-mm’. For each probe set in each cell line, we quantified the change in expression for each time point against time zero as CE_T_ = log_2_ *X*_T_ - log_2_ *X*_0_. To identify probe sets differentially regulated in cells expressing wild-type and mutant LPAR1 receptors, we calculated the differential change as DCE_T_ = CE_mt,T_ - CE_wt,T_, and ranked the probe sets accordingly. To identify pathways associated with the LPAR1 mutant in response to LPA ligand, we applied gene set enrichment analysis on this ranked gene list using the 268 mouse gene sets in MSigDB v3.0 and gene permutations (*n*=10,000) [24]. The full list of gene sets for this analysis is available in Table S5 (1 hour treatment) and Table S6 (2 hours treatment). Expression array data are available from the Gene Expression Omnibus database under accession number GSE34629 at http://www.ncbi.nlm.nih.gov/geo/query/acc.cgi?token=blydzmiaagwcknq&acc=GSE34629 .

### Cell growth assay and migration assays

 NIH 3T3 cells were cultured in media containing 0, 1, and 10% new-born bovine serum (NBS, Sigma), and growth was monitored using Cell TiterGlo proliferation assays (Promega, Madison, WI). Eight replicate wells were used for each condition. Boyden chamber assays were performed as previously described [25], and scratch assays were carried out in full DMEM media containing 10% NBS, 1% P/S, and 0.4 mg/ml of G418. Cells were grown to near confluency before a scratch was made using 200μl pipette tips. Photos were taken after additional 16-18 hours incubation and staining with Diff-Quik (Siemens, Deerfield, IL, USA). For ROCK inhibitor Y27632 (Sigma, St. Louis, MO) experiments, cells were pre-incubated with 20 μM Y27632 or vehicle in DMEM media containing 1% NBS, 1% P/S for 1 hours before scratches. 

### Rho activation assay

Rho activity was measured using a Rho activation assay kit made by Millipore (Damstadt, Germany) according to the manufacturer’s instruction. Briefly, 5 X 10^5^ NIH3T3 cells transfected with wild-type, mutant LPAR1 or vector were cultured in 150 mm cell culture dishes with DMEM media containing 10% NBS (Sigma) for 24 hours; then 1% NBS for another 24 hours; and finally with serum-free DMEM for additional 24 hours (cells reached to approximately 30% confluence). Cells were treated with 10 μM LPA for 0, 5 and 15 minutes before harvested for Rho activation assays. Rho-GTP was pulled down by 40 μg of Rhotekin-agarose beads from 800 µg of cell lysate for each condition. Entire pulled down Rho-GTP was loaded to a 4-20% Tris-glycine polyacrylamide gel (Invitrogen, Carlsbad, CA) for western blotting of GTP-bound Rho. In parallel, 30 µg of total protein lysate was loaded onto another 4-20% Tris-glycine polyacrylamide gel for western blotting of total Rho and β-actin. An anti-β-actin antibody was purchased from Santa Cruz Biotechnology (Dallas, TX). The immunobands were quantified using ImageJ image analysis software (http://rsbweb.nih.gov/ij/), and the GTP-bounded Rho was normalized using total Rho and β-actin.

### Measurement of Rho-Rock signaling using a SRE luciferase reporter

 Fifty nanograms of pSRE luciferase reporter (Stratagene, La Jolla, CA) and 20ng of pRLNull (Promega, Madison, WI) was co-transfected into COS-7 cells with 10 ng of LPAR1 WT or 10 ng of LPAR1 MT construct using Lipofectamine-Plus (Invitrogen, Carlsbad, CA). Cells were kept under normal culture condition for 48 hours and then were serum starved overnight before treated with different concentrations of LPA (Sigma, St. Louis, MO) for 4 hours. Cells were harvested for detection of the luciferase activity using a Dual-Glo Luciferase Assay Kit (Promega, Madison, WI). 

## Results

### Case Presentation

 A 19-year-old female patient presented with a large right-sided adrenal primary tumor with bone and bone marrow metastases. At diagnosis, a pre-chemotherapy bone marrow biopsy was obtained and had >90% tumor infiltration (labeled as Met1). The patient was treated with the standard induction chemotherapy as per the Children’s Oncology Group (COG) high-risk neuroblastoma protocol A3973. After four cycles of induction chemotherapy, there was an improvement in clinical symptoms, but with a mixed radiographic response. At this point the patient underwent a surgical procedure to remove the primary tumor. The bulk primary tumor was macro dissected into 4 different sections for further genomic studies; and they all contained ≥65% viable tumor cells (labeled as PT for Primary Tumor). Despite subsequent multiple rounds of salvage therapy, the patient continued to have progressive disease and developed extensive metastases to lung, bone, brain, and liver. Eventually, the patient succumbed to the disease 3.5 years after the initial diagnosis. A metastatic tumor from liver was taken for genomic analysis (>90% tumor cells; labeled as Met2), while normal skin and unaffected liver were taken as germ line samples. 

### Whole Genome Sequencing of Met2 and germline DNAs

**Figure 1:**
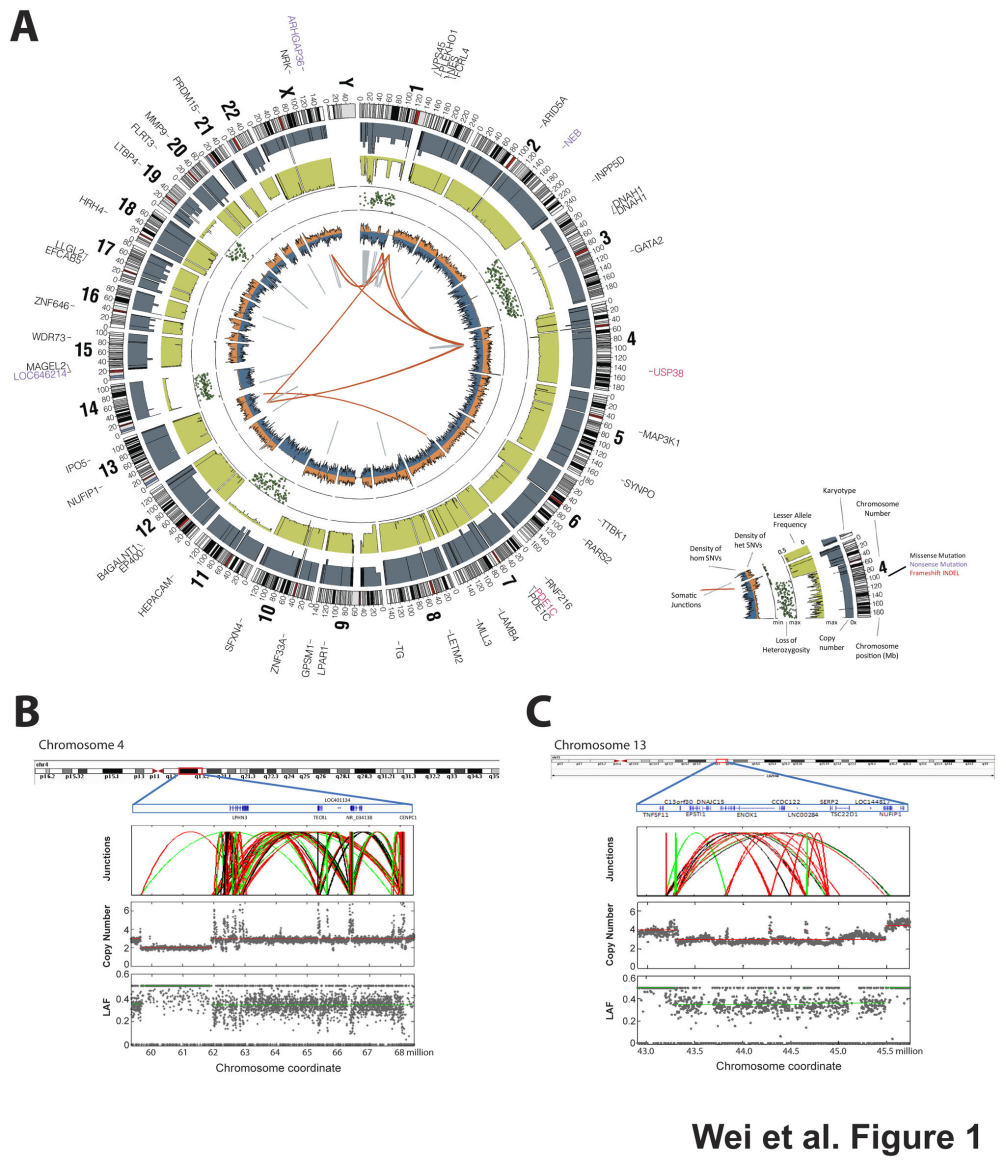
Whole genome sequencing of tumor Met2 and paired germline DNA revealed extensive somatic alterations in tumor DNA. (**A**) A CIRCOS plot shows non-synonymous somatic mutations, copy number changes, lesser allele fraction, loss of heterozygosity (LOH), and abnormal junctions in the Met2 tumor genome. (**B**,**C**) Chromothripsis was evident by massive complex rearrangements detected at chromosomes 4q and 13p by whole genome sequencing. In the junction plots, black, green, and red lines represent deletions, tandem duplication, and inversion respectively. Each dot in the copy number and LAF plots represents a 2 kilobases DNA fragment, and red and green lines mark the average segmental copy number and LAF respectively. LAF, lesser allele fraction.

To identify somatic non-synonymous mutations at the end of disease, we performed whole genome sequencing on the index metastatic tumor Met2 and its paired germline DNA (normal skin) using unchained combinatorial probe anchor ligation sequencing chemistry [21]. Approximately 311 and 317 gigabases (Gb) of mapped sequence was acquired for the germline and tumor genomes (equivalent to average coverage of 106X and 108X respectively). The high sequencing coverage enabled us to call 98.9% of the germline and 98.8% of the tumor genome with at least 20X coverage. Comparing to the human reference genome (hg19), small variant analysis revealed each genome contained 5.05 and 4.77 million small variants for germline and tumor respectively including single nucleotide variants (SNVs), indels (<50bp), and substitutions (Table S1). Comparison of variants from tumor and germline, we identified 61 non-synonymous mutations out of 8508 high-quality somatic small variants in the tumor (somatic scores ≥-10). Forty-four of the 61 non-synonymous mutations were confirmed experimentally using orthogonal re-sequencing methods including Sanger or semiconductor sequencing (Figure 1A and Table S2), corresponding to an accuracy of 72% and a mutation rate of 0.85 per mega bases for somatic non-synonymous mutations, similar to what was reported in neuroblastoma [15,17]. Out of the 44 mutant genes, only 1 gene (*NEB*) was reported to be mutated in other neuroblastoma in a published whole genome sequencing study confirming a low frequency of recurrent mutations in this cancer[15]. All of the somatic mutations were absent in the 1000 genomes (February 2012 release) and human SNP database (dbSNP build 135).

In addition to the small variants, structural variant analysis of whole genome sequencing demonstrated extensive chromosomal structural and copy number alterations involving virtually every chromosome resulting in aneuploid (near-tetraploid) in Met2 (Figure 1A). These changes consisted of gain and loss in the regions commonly seen in neuroblastoma including loss of heterozygosity (LOH) on chromosomes 1p, 3, 11, 14, and 19; gain of 1q, 2, 6, 7, 8, 12, 13, 17, 18, 20, and 22 [26]. Chromothripsis [27] was also detected in this tumor, supported by complex massive rearrangements with only 2 major copy number states on chromosome arms 4p and 13q (135 and 24 high-confidence structural junctions in 8.5Mb and 1.5 Mb respectively) (Figure 1B and C, Table S3). This was in keeping with a recent observation of chromothripsis in 18% of patients with high-stage neuroblastoma [15]. 

**Figure 2:**
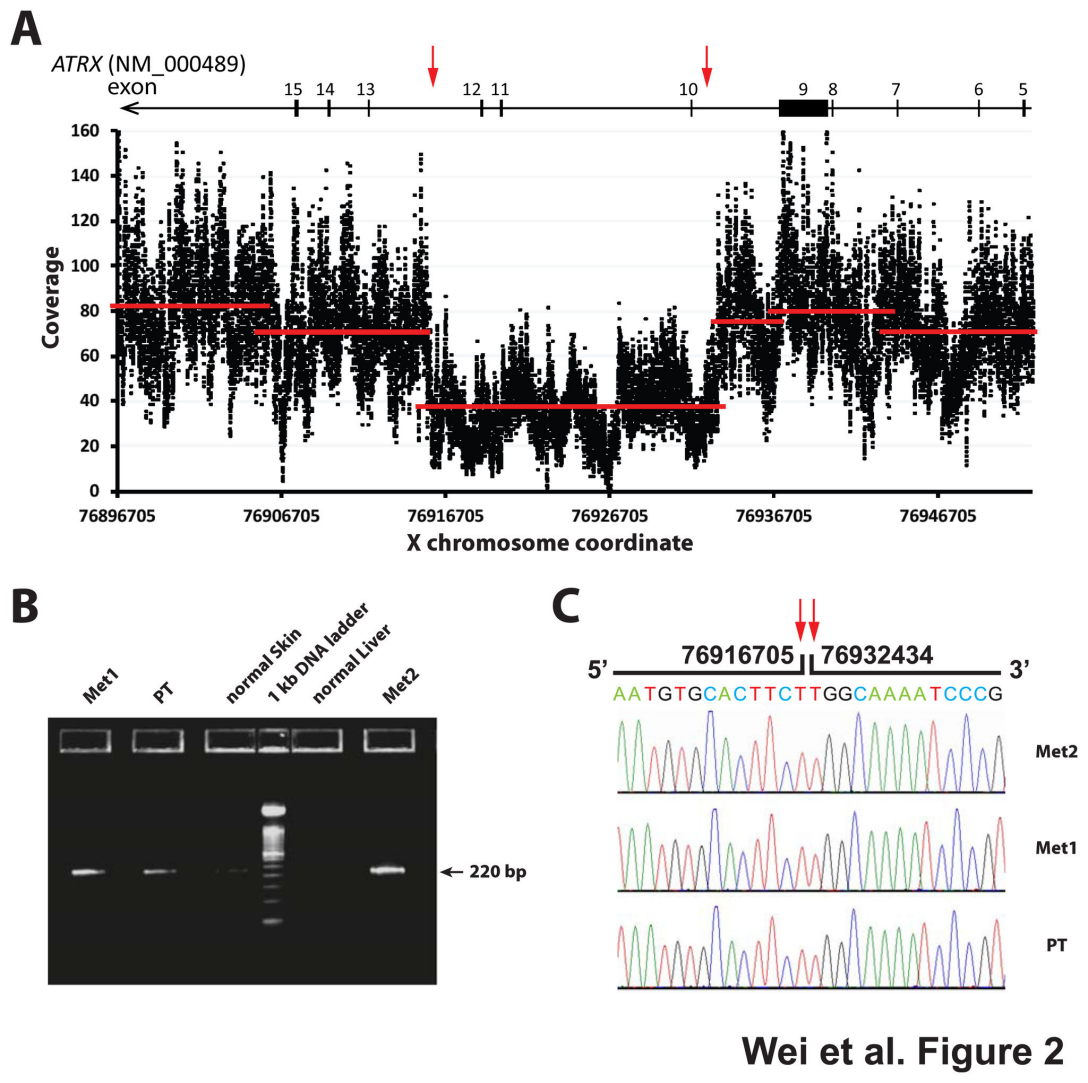
A focal hemizygous deletion in the *ATRX* gene in all three tumor samples. (**A**) Base coverage of X chromosome from whole genome sequencing detected a hemizygous deletion in the *ATRX* genes from 76916706 to 76932433 (marked by red arrows). Each black dot represents a base, and red lines denote average coverage of each segment using circular binary segmentation[67]. Transcript NM_000489 was used for the exon annotation of the *ATRX* gene. (**B**) Genomic PCR verified the somatic deletion in the *ATRX* gene using a pair of genomic PCR primers flanking the predicted deleted region on the X chromosome. As result of the deletion, an abnormal PCR product of 220bp was detected in tumor DNAs, but not in the germline DNAs on a 2% TBE-agarose gel. (**C**) Sanger sequencing of the genomic PCR products confirmed the deletion of ChrX:76916706-76932433 was the same among all three tumors .

Loss-of-function somatic *ATRX* mutations including large deletions was recently reported in neuroblastoma by three independent studies [14,15,17]. While our whole genome sequencing did not detect any small variant mutation, structure analysis identified a focal hemizygous deletion of 16 Kb involving exons 10-12 in the *ATRX* gene in Met2 as shown in a coverage plot at the base-pair level (Figure 2A) and indicated by detection of an abnormal junction in the tumor DNA (Table S3). Using genomic PCR with a pair of primers flanking this deleted region, we experimentally verified the *ATRX* deletion in all three tumor samples (Figure 2B). Sanger sequencing of the PCR products further demonstrated that the in-frame deletions in the *ATRX* gene were exactly the same among three tumors (Figure 2C). The presence of the deletion in the diagnostic tumor sample Met1 and the primary tumor (PT) suggested the importance of this event for tumorigenesis, and this result was also consistent with the finding of frequent *ATRX* mutations in adolescent and young adult patients with neuroblastoma [14].

### Commonly mutated genes among metastatic and primary Tumors

 To determine if the 44 small somatic variant mutations identified in Met2 were present in the prior tumor samples in the history of this patient, we sequenced the genomic DNAs of Met1 (bone marrow acquired at diagnosis) and 4 distinct portions of PT (primary tumor taken at resection) for these mutations using a combination of Sanger and semiconductor sequencing. The latter is a sensitive technique using hundreds or even thousands of reads to detect rare variants with frequency as low as 1%. Fourteen of the 44 small variants (32%) were detected in Met1 and in all the sections of PT, while 30 (68%) were validated but unique to only Met2 (Table S2), indicating that these common mutations arose early and might play roles during initial tumorigenesis. In addition, using ultra deep sequencing with hundreds-thousands reads coverage, the 30 Met2-unique variants were absent in all 4 sections of the primary tumor and Met1 (Table S2), suggesting that these novel mutations occurred *de novo* during the course of the disease possibly related to therapy. The similar frequencies of these mutations in the 4 sections of primary tumor also indicated homogeneity in the primary tumor (Table S2). 

### Expression of mutant alleles among metastatic and primary tumors

**Figure 3:**
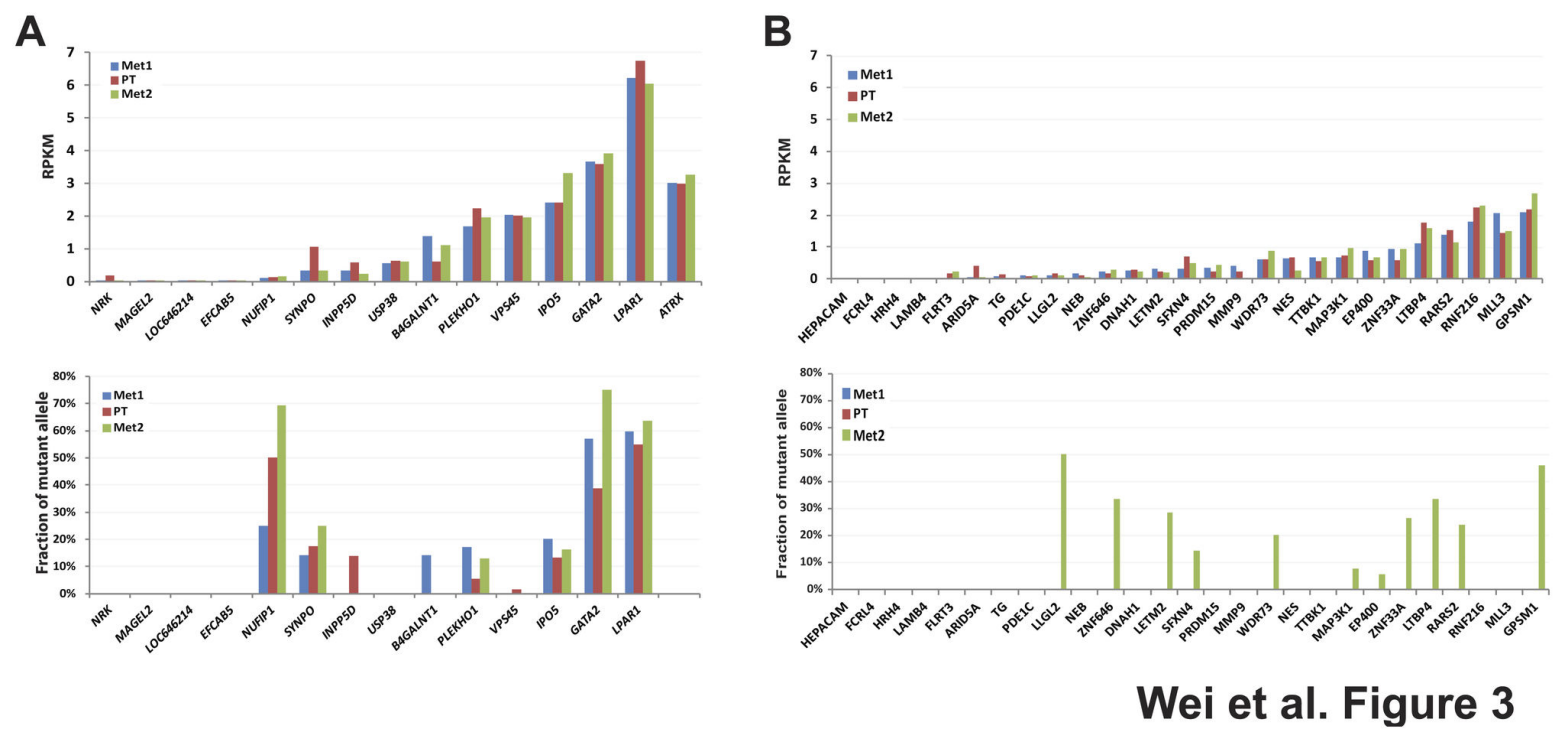
Transcriptome sequencing demonstrated that *LPAR1* was the most highly expressed gene among the 15 commonly mutated genes. (**A**) Expression of 15 commonly mutated genes. Upper panel, normalized expression at the gene level was plotted for the 15 shared mutant genes in reads per kilobases of exon per million mapped reads (RPKM). Most genes were expressed at similar levels among all three tumors and *LPAR1* was the highest expressed mutant gene. Lower panel, fraction of mutant allele transcripts was calculated at the mutant positions for 14 commonly mutated genes. (**B**) Expression of 30 mutated genes unique to Met2. Upper panel, Normalized expression at the gene level in reads per kilobases of exon per million mapped reads (RPKM). Lower panel, fraction of mutant allele transcripts.

To determine which of the somatic mutant variants were expressed and their expression level, we performed transcriptome sequencing on all three tumors (Met1, one of the PTs, and Met2). Gene expression was detected in 12 of the 15 commonly mutated genes (Figure 3A, upper panel), and 9 of the 12 had detectable level of mutant alleles (Figure 3A, lower panel). Only 3 genes (*NUFIP1*, *GATA2*, and *LPAR1*) had high fraction of mutant allele (>30%) in their transcripts (Figure 3A, lower panel), suggesting their potential roles as driver oncogenes. Some of the remaining 30 Met2-only mutant genes also had expression of their mutant alleles, but as expected only restricted to the Met2 (not Met1 or PT, Figure 3B). These expressed somatic mutations may represent potential *de novo* oncogenes arising during the course of the disease.

Of the three commonly expressed mutant genes, *LPAR1* encodes a G-protein coupled receptor for lysophosphatidic acid (LPA), a ubiquitous phospholipid in normal tissues. In this patient, both expression level and the fraction of mutant allele of *LPAR1* was among the highest of the 15 commonly mutated genes (Figure 3A), implying a potential driver oncogene function in this tumor. LPAR1 regulates important cellular processes such as cell proliferation, migration, signaling, neural development, and cell differentiation [28-30]. We sequenced the coding region of *LPAR1* for additional 23 neuroblastoma tumors from patients aged greater than 6-year old including 14 from metastatic high-risk patients (Table S4) using Sanger sequencing method. We did not find any protein-disrupting mutation in our samples (data not shown), neither had the published sequencing studies identified any mutation in the *LAPR1* gene [15-17], suggesting *LPAR1* somatic mutation is unique to this patient. Nevertheless, because *LPAR1* is involved in the RHO pathway and the genes in this pathway have been found to be frequently mutated in neuroblastoma [15], we performed further biological characterization of this somatically acquired mutation.

### Investigation of the molecular effects of the LPAR1 R163W mutation by microarray gene expression profiling

**Figure 4:**
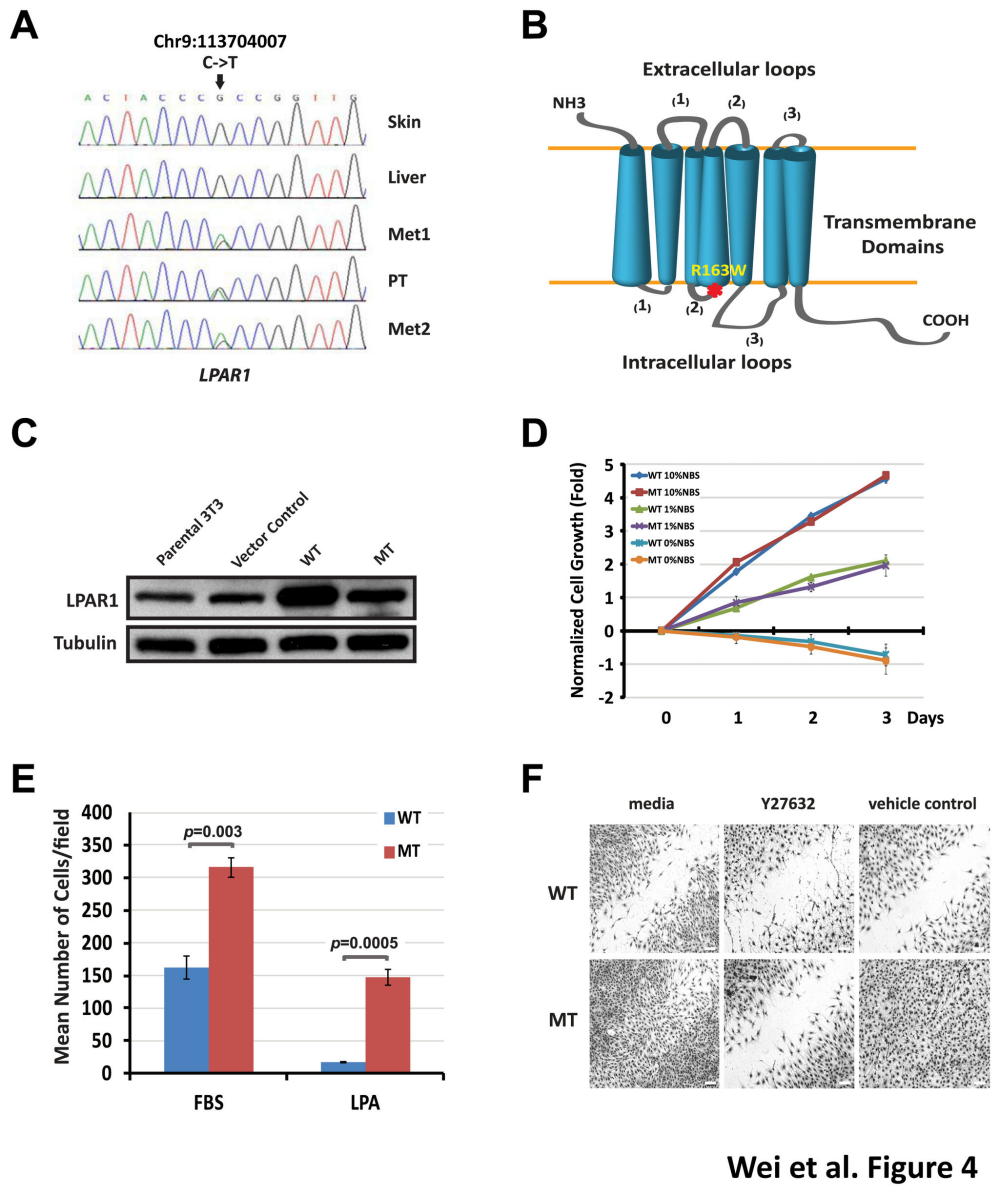
*LPAR1* (R163W) mutation does not affect cell growth, but causes increased cell motility. (**A**) Sanger sequencing validated the *LPAR1* mutation. The mutation was present only in tumors, not in the germ line DNAs. (**B**) A schematic diagram shows the position of the R163W mutation (*) which was located at the junction of the second intracellular domain and fourth transmembrane domain of LPAR1. (**C**) A Western blot showed forced expression of human wild-type (WT), R163W mutant (MT), and mouse endogenous LPAR1 receptors in NIH3T3 cells using a rabbit polyclonal antibody from Novus Biologicals (Littleton, CO). (**D**) NIH3T3 cells expressing mutation (MT) and wild-type of LPAR1 (WT) grew at a similar rate in 0, 1, and 10% new born bovine serum (NBS). Each time point was normalized to day 0, and the average cell growth was plotted. Error bars represent standard deviations. (**E**) NIH3T3 cells expressing MT LPAR1 showed a significant increased motility compared to those expressing WT LPAR1 in response to serum or LPA gradient in a Boyden chamber assay. Error bars represent standard errors. (**F**) Scratch assays confirmed the increased motility of NIH3T3 cells expressing MT compared to WT LPAR1. A Rho kinase (ROCK)-specific inhibitor, Y27632, reduced the cell motility mediated by the MT LPAR1 receptor. White scale bars are 200 μm.

The C to T transition results in a substitution of an arginine to a tryptophan at amino acid 163 (R163W) at the junction between the second intracellular domain and the fourth trans-membrane domain of the LPAR1 receptor (Figure 4A and B), predicted to be damaging by both SIFT [31] and PolyPhen-2 [32] (Table S2). In order to explore the molecular effects mediated by the mutant LPAR1, we compared the gene expression profiles of NIH3T3 cells stably expressing wild-type and mutant human LPAR1 receptors in response to its ligand LPA using microarrays. Forced expression of human LPAR1 was confirmed by a Western blot to ensure a comparable expression of wild-type and mutant in NIH3T3 cells (Figure 4C). In a gene set enrichment analysis for cells expressing mutant LPAR1, we found a significant enrichment of differentially expressed transcripts associated with haptotaxis or cell motility in response to migratory stimuli [33] at both 1 and 2 hour time points in cells expressing mutant LPAR1 (Table 1, Table S5, and S6). Additionally, an enrichment of genes induced by RhoA [34] suggested activation of Rho pathway (Table 1). There was no enrichment of cell cycle or growth related gene set. Therefore, these results predicted that the LPAR1 R163W mutation would result in an increased mobility phenotype *via* the RHO pathway without impacting the cell growth rate.

**Table 1:** Gene set enrichment analysis of the change of gene expression profiles in response to lysophosphatidic acid (LPA) (FDR<0.01).

**Gene Set ID**	**Size**	**NES**	***P*-value**	**FDR**
***Gene sets enriched at 1 hour***				
MILI_PSEUDOPODIA_HAPTOTAXIS_UP	408	3.3	<1.0E-04	<1.0E-04
FOSTER_INFLAMMATORY_RESPONSE_LPS_DN	316	2.9	<1.0E-04	5.5E-04
BERENJENO_TRANSFORMED_BY_RHOA_UP	435	2.8	<1.0E-04	8.7E-04
MONNIER_POSTRADIATION_TUMOR_ESCAPE_UP	248	2.6	<1.0E-04	3.4E-03
***Gene sets enriched at 2 hours***				
MILI_PSEUDOPODIA_HAPTOTAXIS_UP	408	3.0	<1.0E-04	5.0E-04
MONNIER_POSTRADIATION_TUMOR_ESCAPE_UP	248	2.8	1.0E-04	2.7E-03

NES, normalized enrichment score. FDR, false discovery rate.

### Characterization of the LPAR1 mutation in-vitro

**Figure 5:**
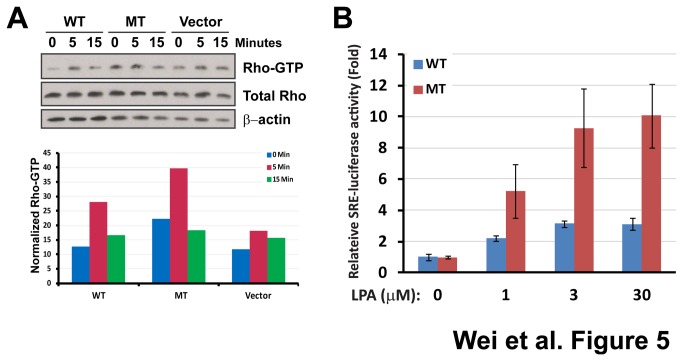
*LPAR1* (R163W) mutation promotes cell mobility through activation of Rho pathway. (**A**) Rho activation assays (upper panel) showed a transient increase of GTP-bound Rho after exposure to LPA ligand (10 μM) in both wild-type (WT) and mutant (MT) LPAR1 expressing NIH3T3 cells indicating activation of Rho pathway mediated by the receptors. Signal of immunobands in the Western blot (upper panel) was quantified, and abundance of the GTP-bound Rho is plotted after normalization against total Rho and β-actin in the lower panel. (**B**) MT LPAR1 showed heightened signaling through the Rho pathway in transiently transfected COS-7 cells exposed to LPA in a dose-response way (*P*<0.05) compared to WT. SRE represents the serum response element activity normalized against renilla luciferase and vehicle control. Error bars represent standard errors.

When we measured the growth rate of NIH3T3 cells stably expressing wild-type and mutant LPAR1, there was indeed no significant difference of growth in culture media containing 0, or 1%, or 10% serum, confirming the microarray finding (Figure 4D). However, we observed a significant increase of motility for cells expressing mutant LPAR1 compared to cells expressing wild-type LPAR1 in a trans-well migration assay using Boyden chambers (*P*<0.01; Figure 4E) and in scratch assays (Figure 4F). Cells expressing wild-type LPAR1 had a similar motility as the parental or vector-control cells (Figure S2), signifying that the increased motility was a result of the mutation. Furthermore, the increased motility in NIH3T3 cells expressing mutant LPAR1 receptor could be abolished by pre-incubating cells with a ROCK inhibitor Y27632 in the scratch assays (Figure 4F), suggesting activation of Rho-ROCK pathway by the mutant LPAR1 receptor. This result was consistent with the expression profiling experiments which predicted activation of Rho pathway in NIH3T3 cells expressing mutant LPAR1 receptors. The RHO-ROCK pathway has been reported to regulate cell motility [35,36] and is one of canonical pathways downstream of LPAR1. We therefore investigated if the mutation had any impact on this pathway using a Rho activation assay in NIH3T3 cells and a luciferase reporter assay containing serum response elements (SRE, a downstream target of Rho-ROCK signaling) in COS-7 cells. Rho activation assays showed a transient increase of GTP-bound form of Rho after exposure to LPA ligand in both wild-type and mutant LPAR1 expressing NIH3T3 cells indicating the activation of Rho pathway mediated by LPAR1 receptors (Figure 5A). As expected, the mutant LPAR1 receptor demonstrated a higher activity than the wild-type receptor (Figure 5A). Furthermore, luciferase assays in transient transfected COS-7 cells showed that both wild-type and mutant LPAR1 receptors responded to their ligand LPA in a dose-dependent fashion; and there was a significantly enhanced SRE response to LPA in cells expressing the mutant receptor (Figure 5B). Therefore, both experiments clearly demonstrated that the R163W mutation of LPAR1 increases cell motility through augmented signaling of the RHO-ROCK pathway.

## Discussion

High resolution methods such as genome-wide sequencing have revealed complex intra- and inter-tumor heterogeneity of cancer [37,38]. We sequenced three tumors taken at different time throughout the disease course of a patient, and found a core set of 15 shared mutations persistently presented during the full course of the disease. We further have shown that some of these 15 shared mutated genes expressed the mutant transcripts possibly functioning as driver oncogenes. In addition to the shared mutations, thirty other mutations arose in a liver metastasis that were not detected either in the bone marrow metastasis at diagnosis or in any of four distinct parts from the primary tumor using ultra-deep sequencing. Different from the published studies demonstrating intra-tumor heterogeneity of the primary tumors [37,38], the similar frequencies of all variants among different sections of the primary tumor implied a surprisingly consistent mutation profile and absence of a tumor clone resembling the liver metastasis in the primary tumor in our study. Therefore, although the possibility of complete elimination of Met2-like cancer cells from the primary tumor by therapies cannot be ruled out since the primary tumor was taken after treatment, these 30 mutations were likely *de novo* mutations acquired during 3.5 years’ treatment. Interestingly *ARID5A* was among these 30 *de novo* mutant genes unique to Met2, and a recent sequencing study has reported that mutations in the chromatin-remodeling genes *ARID1A* and *ARID1B* in neuroblastoma were associated with a more aggressive phenotype [16]. The function of these *de novo* mutations is currently unknown, but they may represent either genes involved in tumor progression or chemo-resistance or possibly passenger mutations. Nevertheless, the genetic heterogeneity of the three tumors in our study strongly suggests that detailed genomic analyses of several tumor samples and repeat biopsies during the course of a progressive disease are indicated, although sequential biopsies are not currently performed as part of the management of patients with progressive disease. 

A systematic approach to identify druggable targets is not only necessary for targeted therapy in cancer patients but also crucial for understanding cancer biology. Genomic sequencing efforts in cancer have identified numerous somatic non-synonymous mutations; however, it is not clear whether putative mutant alleles have biological consequence or are even expressed. Furthermore, cancers like neuroblastoma have surprisingly few recurrent mutations in recent genomic studies of neuroblastoma using massively parallel sequencing [14-17]. We only identified 2 mutated genes (*ATRX* and *NEB*) in this neuroblastoma patient that have been reported in the previous studies. Therefore the heterogeneous nature of neuroblastoma genome and the rarity of the recurrent mutations will make it challenging to identify druggable targets in an individual patient for treatment. Here, we demonstrated a systematic approach to identify driver oncogenes by sequencing both tumor genomic DNA and transcriptome in multiple tumor samples from the same patient (Figure S1).

Mutations in tumor suppressor genes usually result in loss of expression such as in nonsense-mediated decay of their mutation-harboring messenger RNAs. Therefore, restoration of a mutant tumor suppressor gene is much more challenging than targeting a driver oncogene. For example, *ATRX*, a chromatin remodeling gene on the X chromosome, is a putative tumor suppressor of which loss-of-function mutations are strongly correlated with alternative lengthening of telomeres (ALT, a mechanism independent of telomerase activity) in cancers [39-41]. Three recent studies reported that mutations in *ATRX* often resulted in loss of expression in neuroblastoma, and Cheung et al further showed that mutations of *ATRX* were more prevalent in older and stage IV neuroblastoma patients [14,15,17]. Consistent with these studies, we detected a large hemizygous deletion predicting an in-frame deletion of exons 10-12 in the *ATRX* gene in all three tumors from this young adult patient. Therefore, *ATRX* mutations and the downstream deregulated ALT may provide a key to understand the tumor biology and an opportunity for novel therapies in the late onset neuroblastoma patients. Because of the loss-of-function of *ATRX* mutations, direct restoration of *ATRX* normal function may be difficult as a novel therapy. However other molecules along this pathway may be better alternative targets for treatment. 

Gain-of-function mutations are often the hallmark of an oncogene. The mutant alleles of driver oncogenes would be expected to be expressed to exert their oncogenic functions, and inhibition of oncogenes is routinely achieved in clinic with pharmaceutical agents [42-44]. In order for a mutant allele to be a driver oncogene, it is required to be expressed. Recent studies have highlighted the importance of expression of mutant genes for identification of drivers in other cancers [45-47]. We therefore attempted to identify driver expressed oncogenes by performing genomic and transcriptome sequencing in parallel. We first identified 15 somatically mutated genes present in all three tumor samples taken at different time points during the treatment which were candidate cancer driver genes. We then examined the expression fraction of the mutant alleles in data from transcriptome sequencing. Only three genes (*NUFIP1*, *GATA2*, and *LPAR1*) of the 15 commonly mutated genes had both detectable transcripts and high mutant allele fractions (>30%) in their transcripts. The remaining genes of low variant allele expression (<30%) were unlikely to be driver oncogenes, but they may represent possible tumor suppressor genes.

Of the highly expressed mutant genes, human fragile X mental retardation interacting protein gene *NUFIP1* was reported to interact with *BRCA1* and maintain genome stability in yeast [48,49]. Neurological abnormality in patients with deletions in *NUFIP1* suggests its important role in normal neuron development [50,51]. Of note, both ATRX and NUFIP1 physically interacts with BRCA1 [48,52], suggesting their possible functions in the same protein complex. *GATA2* is a transcription factor necessary for normal hematopoietic and neural development [53-55], and is often specifically and highly expressed in neuroblastoma [56,57]. Loss-of-function *GATA2* mutations have been reported in patients with familial myelodysplastic syndrome and acute myeloid leukemia (MDS/AML) [58,59], but *GATA2* mutations have not been reported in neuroblastoma. The *GATA2* mutation identified in this study is different from those commonly mutated in the MDS/AML, and its biological consequence needs to be characterized in the future studies. 

Mutations of LPAR1 have been identified in lung and liver cancers in rat models exposed to carcinogen [60,61]. However, to our knowledge, this is the first report of a human *LPAR1* mutation having biological consequence in a human cancer. Lysophosphatidic acid (LPA) is a known chemotactic molecule for cells [30] and wild-type Lysophosphatidic acid receptor 1 (LPAR1) has been shown to increase cell motility, invasion and metastasis in breast, liver, lung and colon cancers [25,62-66]. In this study, we sequenced the *LPAR1* coding region in additional 23 neuroblastoma cancers including 14 metastatic high-risk tumors, but did not find any non-synonymous mutation in these samples. In addition, recent sequencing analyses on neuroblastoma also did not identify any mutation in *LPAR1* [15-17], suggesting the mutation in this gene is rare in neuroblastoma. However, one major finding in one of the reports was frequent activation of the RHO signaling pathway by mutations to inhibit neuritogenesis in neuroblastoma [15], and LPAR1 is a membrane receptor in this pathway. Here we demonstrated that the LPAR1 R163W mutation preferentially affected cell motility over growth through augmented RHO-ROCK signaling. Therefore, our study indicates and confirms that the activation of the RHO pathway may play an important role in neuroblastoma, and the shows importance to detect extremely rare mutations for precision therapy for patients with cancers lacking recurrent mutations. 

 In conclusion, sequencing of an index metastatic tumor from a neuroblastoma patient showed evidence for massive chromosomal alterations together with a small set of somatically acquired expressed mutations that happened early during the tumorigenesis. Furthermore, examination of these somatic mutations in additional tumor samples revealed accumulation of *de novo* mutations during therapy. Parallel whole genome and transcriptome sequencing identified a cell motility driver mutation in the *LPAR1* gene, and this combinatorial approach may be leveraged for precision therapy in patients with cancer by targeting expressed driver mutations. 

## Supporting Information

Figure S1 **Flow diagram of next-generation sequencing experiments to identify expressed driver mutations in a patient with metastatic neuroblastoma.**
(PDF)

Figure S2 **Parental and vector-transfected NIH3T3 cells have a similar motility rate as the cells expressing wild-type LPAR1 in a scratch assay.**
(PDF)

Table S1 **Statistics of whole genome sequencing.**
(PDF)

Table S2 **Validated 44 somatic non-synonymous mutations in Met2.**
(PDF)

Table S3 **High-confidence junctions.**
(PDF)

Table S4 **Clinical informaiton of 23 nueroblastoma patients.**
(PDF)

Table S5 **Gene set enrichment analysis of the change of gene expression profiles in response to LPA (1 hour).**
(PDF)

Table S6 **Gene set enrichment analysis of the change of gene expression profiles in response to LPA (2 hours).**
(PDF)
