# Supplementary material for: Massively Parallel Sequencing Reveals an Accumulation of De Novo Mutations and an Activating Mutation of LPAR1 in a Patient with Metastatic Neuroblastoma
Source: PLoS One. 2013 Oct 16;8(10):e77731. doi: 10.1371/journal.pone.0077731 (PMC3797724; doi:10.1371/journal.pone.0077731)
Supplement: Figure S1 — Flow diagram of next-generation sequencing experiments to identify expressed driver mutations in a patient with metastatic neuroblastoma. (PDF) [file pone.0077731.s001.pdf]

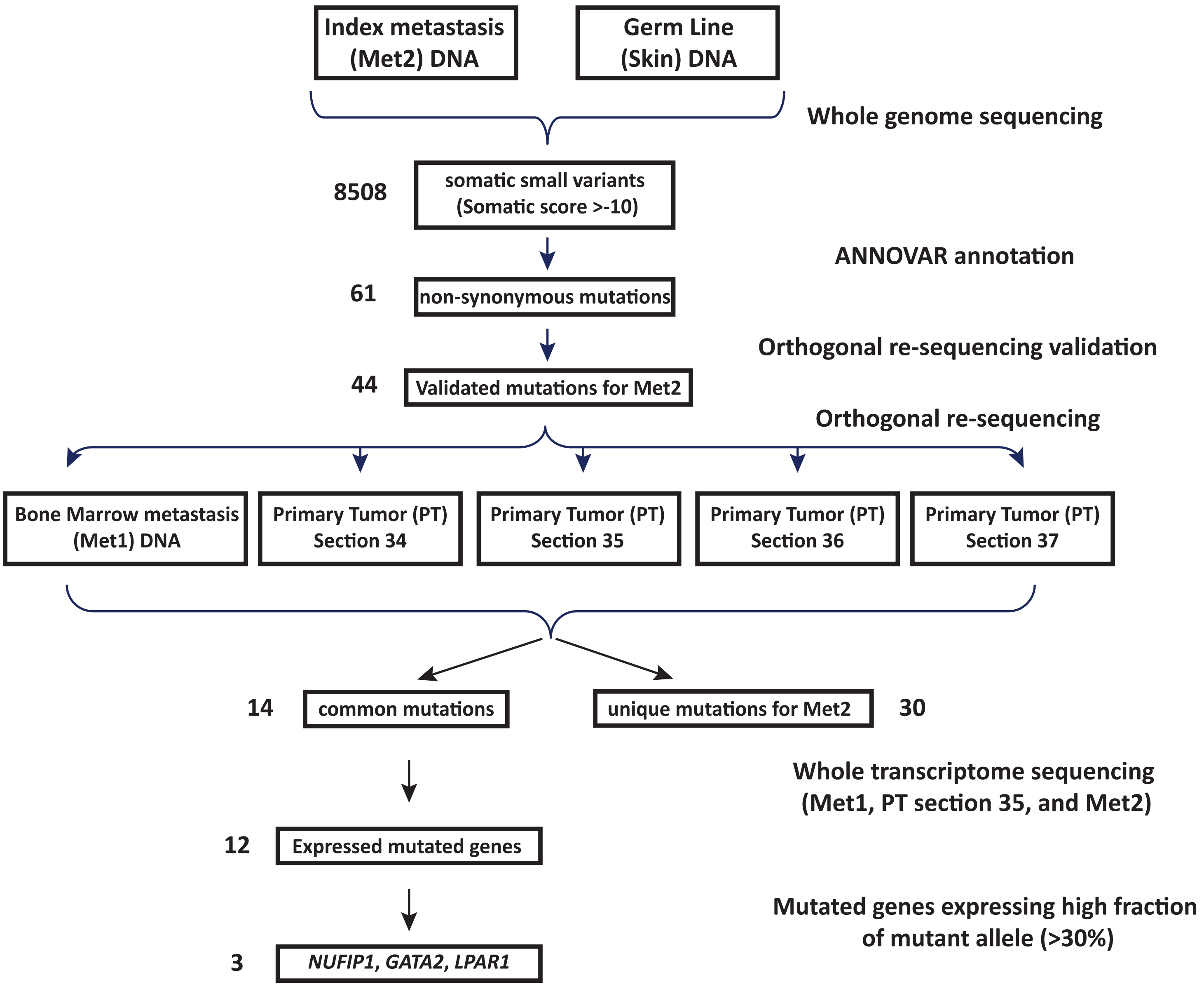

Figure S1. Flow diagram of next-generation sequencing experiments to identify expressed driver mutations in a patient with metastatic neuroblastoma. The numbers beside each box indicate number of small variants. Orthogonal re-sequencing includes Sanger and semiconductor sequencing.
