# Supplementary material for: Massively Parallel Sequencing Reveals an Accumulation of De Novo Mutations and an Activating Mutation of LPAR1 in a Patient with Metastatic Neuroblastoma
Source: PLoS One. 2013 Oct 16;8(10):e77731. doi: 10.1371/journal.pone.0077731 (PMC3797724; doi:10.1371/journal.pone.0077731)
Supplement: Figure S2 — Parental and vector-transfected NIH3T3 cells have a similar motility rate as the cells expressing wild-type LPAR1 in a scratch assay. (PDF) [file pone.0077731.s002.pdf]

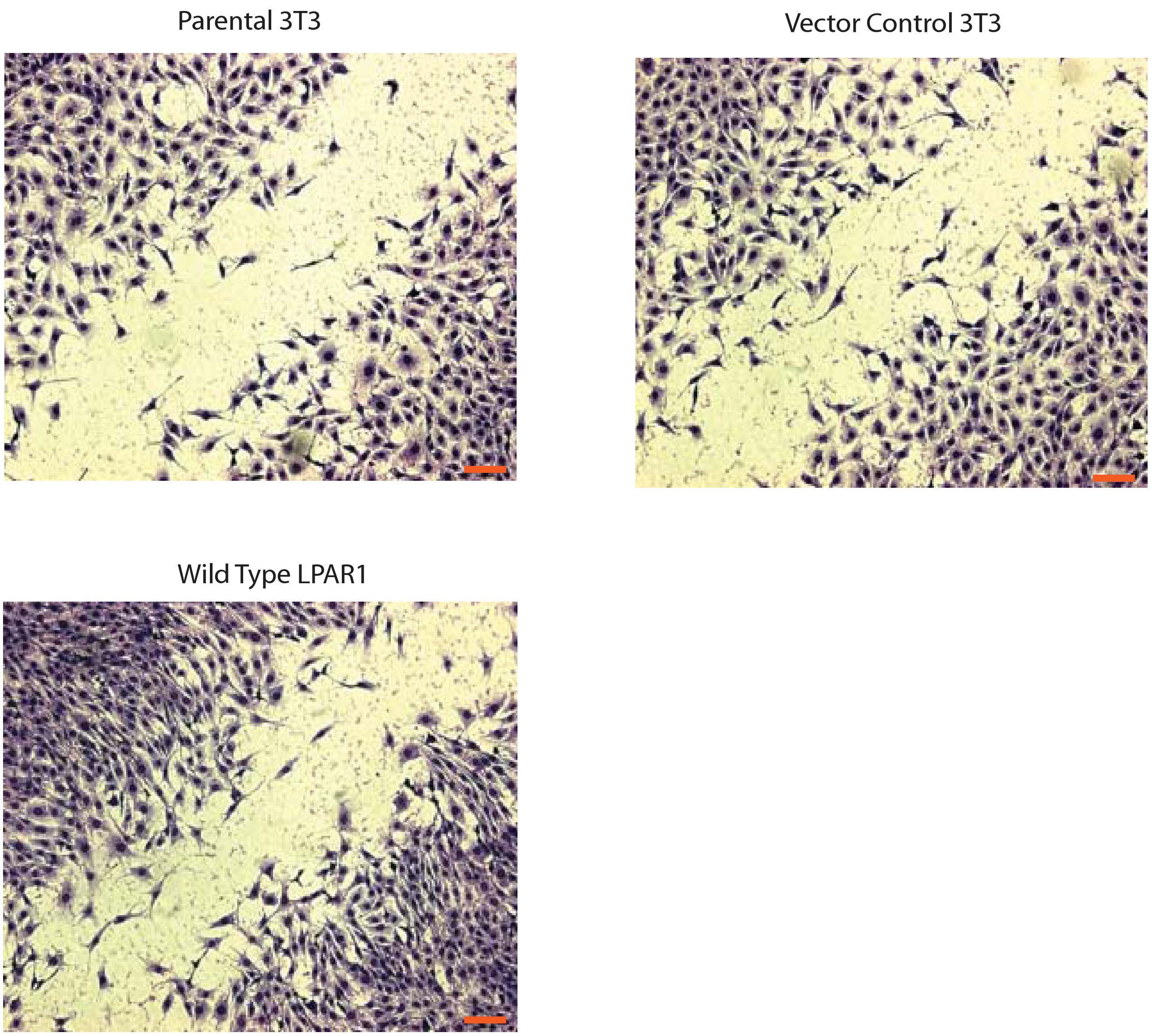

Figure S2. Parental and vector-transfected NIH3T3 cells have a similar motility rate as the cells expressing wild-type LPAR1 in a scratch assay. Red scale bars are 200 μm.
