# Supplementary material for: Massively Parallel Sequencing Reveals an Accumulation of De Novo Mutations and an Activating Mutation of LPAR1 in a Patient with Metastatic Neuroblastoma
Source: PLoS One. 2013 Oct 16;8(10):e77731. doi: 10.1371/journal.pone.0077731 (PMC3797724; doi:10.1371/journal.pone.0077731)
Supplement: Table S1 — Statistics of whole genome sequencing. (PDF) [file pone.0077731.s003.pdf]

Table S1. Statistics of whole genome sequencing

	Met 2	Normal Skin
Total sequence (Gb)	377.6	376.8
Total mapped base (Gb)	316.5	310.8
Average coverage (X)	108.1	106.2
Fraction of genome with ≥20X coverage	98.80%	98.90%
Number of variants called (million)	4.77	5.05
SNV (million)	4.01 (7.3% novel)	4.26 (7.2% novel)
Insertion (million)	0.31 (30% novel)	0.32 (30% novel)
Deletion (million)	0.35 (34% novel)	0.36 (36% novel)
Substitution(million)	0.10 (41% novel)	0.11 (40% novel)
