# Supplementary material for: Massively Parallel Sequencing Reveals an Accumulation of De Novo Mutations and an Activating Mutation of LPAR1 in a Patient with Metastatic Neuroblastoma
Source: PLoS One. 2013 Oct 16;8(10):e77731. doi: 10.1371/journal.pone.0077731 (PMC3797724; doi:10.1371/journal.pone.0077731)
Supplement: Table S2 — Validated 44 somatic non-synonymous mutations in Met2. (PDF) [file pone.0077731.s004.pdf]

Table S2. Validated 44 somatic non-synonymous mutations in Met2

Gene	chr	begin	end	cytoBand	zygosity	somaticCategory	reference	variant	a.a. change	variant type	SIFT_Prediction	SIFT_Score	pph2_predictior	pph2_class	pph2_prob	pph2_FPR	pph2_TPR	pph2_FDR	Variant allele frq_Met1	Variant allele frq_PT_34	Variant allele frq_PT_35	Variant allele frq_PT_36	Variant allele frq_PT_37	Variant allele frq_Met2	Variant allele frq_Normal Skin	Variant allele frq_Normal Liver	Coverage at the variant	Sequencing Methods	Shared (PT&Met2&Met1)
VPS45	1	150064139	150064140	1q21.2	het-ref	snp	G	C	G405A	nonsynonymous	TOLERATED	0.7	benign	neutral	0	1	1	0.575	15%	15%	16%	14%	15%	19%	0%	0%	1000s	Ion Torrent	Yes
PLEKHO1	1	150131220	150131221	1q21.2	het-ref	snp	G	C	E245Q	nonsynonymous	TOLERATED	0.31	benign	neutral	0.361	0.108	0.898	0.14	G/C	G/C	G/C	G/C	G/C	G/C	G	G	1 or 2	Sanger	Yes
INPP5D	2	234070455	234070456	2q37.1	het-ref	snp	C	T	S251L	nonsynonymous	TOLERATED	1							29%	24%	25%	16%	19%	35%	1%	0%	100s	Ion Torrent	Yes
GATA2	3	128199937	128199938	3q21.3	het-ref	snp	G	T	P456Q	nonsynonymous	TOLERATED	1							9%	74%	88%	63%	74%	90%	0%	0%	10s	Ion Torrent	Yes
USP38	4	144135650	144135654	4q31.21	het-ref	del	TATT		f.s.	frameshift_deletion									80%	18%	24%	10%	12%	37%	0%	0%	100s	Ion Torrent	Yes
SYNPO	5	150028224	150028225	5q33.1	het-ref	snp	C	T	P374S	nonsynonymous	TOLERATED	0.16	benign	neutral	0.002	0.704	0.987	0.452	C/T	C/T	C/T	C/T	C/T	C/T	C	C	1 or 2	Sanger	Yes
LPAR1	9	113704006	113704007	9q31.3	het-ref	snp	G	A	R163W	nonsynonymous	DAMAGING	0	probably damaging	deleterious	1	0.00026	0.00018	0.0109	G/A	G/A	G/A	G/A	G/A	G/A	G	G	1 or 2	Sanger	Yes
B4GALNT1	12	58024081	58024082	12q14.1	het-ref	snp	C	T	V189M	nonsynonymous	TOLERATED	0.06	probably damaging	deleterious	1	0.00026	0.00018	0.0109	C/T	C/T	C/T	C/T	C/T	C/T	C	C	1 or 2	Sanger	Yes
NUFIP1	13	45533548	45533549	13q14.12	het-ref	snp	C	G	D330H	nonsynonymous	TOLERATED	1							15%	18%	18%	17%	15%	19%	0%	0%	1000s	Ion Torrent	Yes
IPO5	13	98645247	98645248	13q32.2	het-ref	snp	A	T	T276S	nonsynonymous	TOLERATED	0.73							A/T	A/T	A/T	A/T	A/T	A/T	A	A	1 or 2	Sanger	Yes
LOC646214	15	21935022	21935023	15q11.2	het-ref	snp	G	T	S1906X	stopgain	Not scored	NA							16%	8%	11%	8%	8%	10%	1%	1%	1000s	Ion Torrent	Yes
MAGEL2	15	23892045	23892046	15q11.2	het-ref	snp	G	T	P282T	nonsynonymous	Not scored	NA							51%	29%	29%	24%	27%	28%	0%	0%	1000s	Ion Torrent	Yes
EFCAB5	17	28407946	28407947	17q11.2	het-ref	snp	G	A	R1125K	nonsynonymous	DAMAGING	0	probably damaging	deleterious	1	0.00026	0.00018	0.0109	17%	3%	4%	1%	4%	10%	0%	0%	100s	Ion Torrent	Yes
NRK	X	105137828	105137829	Xq22.3	het-ref	snp	T	A	V128E	nonsynonymous	DAMAGING	0	probably damaging	deleterious	1	0.00026	0.00018	0.0109	T/A	T/A	T/A	T/A	T/A	T/A	T	T	1 or 2	Sanger	Yes
NES	1	156646973	156646974	1q23.1	het-ref	snp	G	T	A28E	nonsynonymous	TOLERATED	0.06	probably damaging	deleterious	0.997	0.0167	0.409	0.0357	G	G	G	G	G	G/T	G	G	1 or 2	Sanger	No
FCRL4	1	157557143	157557144	1q23.1	het-ref	snp	C	T	G257R	nonsynonymous	DAMAGING	0	probably damaging	deleterious	0.995	0.0277	0.681	0.0521	0%	0%	0%	0%	0%	27%	0%	0%	1000s	Ion Torrent	No
ARID5A	2	97215082	97215083	2q11.2	het-ref	snp	G	A	R49Q	nonsynonymous	TOLERATED	0.6	benign	neutral	0.005	0.26	0.968	0.267	1%	0%	0%	0%	0%	60%	0%	0%	1000s	Ion Torrent	No
NEB	2	152551122	152551123	2q23.3	het-ref	snp	C	T	W565X	stopgain	DAMAGING	0.01	probably damaging	deleterious	1	0.00026	0.00018	0.0109	0%	0%	0%	0%	0%	32%	0%	0%	1000s	Ion Torrent	No
DNAH1	3	52380685	52380686	3p21.1	het-ref	snp	C	T	L619F	nonsynonymous	TOLERATED	0.15	benign	neutral	0.054	0.164	0.94	0.191	0%	0%	0%	0%	0%	45%	0%	0%	1000s	Ion Torrent	No
DNAH1	3	52380692	52380693	3p21.1	het-ref	snp	G	T	S621I	nonsynonymous	TOLERATED	0.51	benign	neutral	0.002	0.704	0.987	0.452	0%	0%	0%	0%	0%	45%	0%	0%	1000s	Ion Torrent	No
MAP3K1	5	56177866	56177867	5q11.2	het-ref	snp	C	A	T947K	nonsynonymous	; *Warning! Low con	0.05	benign	neutral	0.002	0.704	0.987	0.452	0%	0%	0%	0%	0%	32%	0%	0%	1000s	Ion Torrent	No
TTBK1	6	43226894	43226895	6p21.1	het-ref	snp	C	G	S379C	nonsynonymous	TOLERATED	0.06	possiblydamaging	deleterious	0.761	0.0768	0.852	0.109	0%	0%	0%	0%	0%	29%	0%	0%	100s	Ion Torrent	No
RARS2	6	88240580	88240581	6q15	het-ref	snp	T	G	E231A	nonsynonymous	TOLERATED	1							0%	0%	0%	0%	0%	27%	0%	0%	1000s	Ion Torrent	No
RNF216	7	5778927	5778928	7p22.1	het-ref	snp	T	A	K367M	nonsynonymous	TOLERATED	1							0%	0%	0%	0%	0%	28%	0%	0%	1000s	Ion Torrent	No
PDE1C	7	31848692	31848693	7p14.3	het-ref	del	C		f.s.	frameshift_deletion									0%	0%	0%	0%	0%	28%	0%	0%	100s	Ion Torrent	No
PDE1C	7	32209549	32209550	7p14.3	het-ref	snp	C	T	C52Y	nonsynonymous	; *Warning! Low con	0							0%	0%	0%	0%	0%	26%	0%	0%	1000s	Ion Torrent	No
LAMB4	7	107735684	107735685	7q31.1	het-ref	snp	G	C	C486W	nonsynonymous	N/A	N/A							0%	0%	0%	0%	0%	14%	0%	0%	1000s	Ion Torrent	No
MLL3	7	151845517	151845518	7q36.1	het-ref	snp	G	T	D4498E	nonsynonymous	DAMAGING	0.02	probably damaging	deleterious	1	0.00026	0.00018	0.0109	0%	0%	0%	0%	0%	22%	0%	0%	1000s	Ion Torrent	No
LETM2	8	38250397	38250398	8p12	het-ref	snp	A	T	E82V	nonsynonymous	TOLERATED	1							0%	0%	0%	0%	0%	19%	0%	0%	100s	Ion Torrent	No
TG	8	134024237	134024238	8q24.22	het-ref	snp	G	T	A2119S	nonsynonymous	DAMAGING	0.02	probably damaging	deleterious	0.974	0.0438	0.763	0.0722	0%	0%	0%	0%	0%	28%	0%	0%	1000s	Ion Torrent	No
GPSM1	9	139252574	139252575	9q34.3	het-ref	snp	G	A	R135H	nonsynonymous	DAMAGING	0	probably damaging	deleterious	1	0.00026	0.00018	0.0109	G	G	G	G	G	G/A	G	G	1 or 2	Sanger	No
ZNF33A	10	38344246	38344247	10p11.21	het-ref	snp	G	T	A399S	nonsynonymous	TOLERATED	0.77	probably damaging	deleterious	0.967	0.046	0.773	0.0746	G	G	G	G	G	G/T	G	G	1 or 2	Sanger	No
SFXN4	10	120917237	120917238	10q26.11	het-ref	snp	T	G	K160T	nonsynonymous	TOLERATED	0.61							0%	0%	0%	0%	0%	39%	0%	0%	1000s	Ion Torrent	No
HEPACAM	11	124791305	124791306	11q24.2	het-ref	snp	G	A	P327S	nonsynonymous	TOLERATED	0.47	benign	neutral	0	1	1	0.575	no coverage	0%	0%	0%	0%	91%	0%	0%	10s	Ion Torrent	No
EP400	12	132466066	132466067	12q24.33	het-ref	snp	A	T	H490L	nonsynonymous	TOLERATED	1	benign	neutral	0.001	0.852	0.994	0.514	0%	0%	0%	0%	0%	16%	0%	0%	1000s	Ion Torrent	No
WDR73	15	85188896	85188897	15q25.2	het-ref	snp	C	G	V230L	nonsynonymous	DAMAGING	0.01	probably damaging	deleterious	0.979	0.0411	0.755	0.0687	0%	0%	0%	0%	0%	72%	0%	0%	1000s	Ion Torrent	No
ZNF646	16	31091627	31091628	16p11.2	het-ref	snp	G	T	C1328F	nonsynonymous	DAMAGING	0	probably damaging	deleterious	0.998	0.0112	0.273	0.0274	G	G	G	G	G	G/T	G	G	1 or 2	Sanger	No
LLGL2	17	73564892	73564893	17q25.1	het-ref	snp	C	A	P432Q	nonsynonymous	DAMAGING	0.02	probably damaging	deleterious	0.992	0.0314	0.704	0.0568	0%	0%	1%	1%	1%	76%	1%	0%	100s	Ion Torrent	No
HRH4	18	22040745	22040746	18q11.2	het-ref	snp	A	T	L18F	nonsynonymous	TOLERATED	1							0%	0%	0%	0%	0%	21%	0%	0%	1000s	Ion Torrent	No
LTBP4	19	41105140	41105141	19q13.2	hom	snp	G	C	G19R	nonsynonymous	N/A	N/A	possiblydamaging	deleterious	0.651	0.0877	0.866	0.121	0%	0%	0%	1%	0%	93%	0%	0%	1000s	Ion Torrent	No
FLRT3	20	14307153	14307154	20p12.1	het-ref	snp	C	A	M333I	nonsynonymous	TOLERATED	0.51	benign	neutral	0.409	0.104	0.894	0.136	0%	0%	0%	0%	0%	21%	0%	0%	1000s	Ion Torrent	No
MMP9	20	44640897	44640898	20q13.12	het-ref	snp	G	A	A374T	nonsynonymous	DAMAGING	0.05	probably damaging	deleterious	0.998	0.0112	0.273	0.0274	0%	0%	0%	0%	0%	24%	0%	0%	1000s	Ion Torrent	No
PRDM15	21	43267231	43267232	21q22.3	het-ref	snp	A	C	S240A	nonsynonymous	TOLERATED	0.09	possiblydamaging	deleterious	0.816	0.0707	0.84	0.102	0%	0%	0%	0%	0%	21%	0%	0%	100s	Ion Torrent	No
ARHGAP36	X	130218251	130218252	Xq26.1	het-ref	snp	C	T	Q207X	stopgain	N/A	N/A							0%	0%	0%	0%	0%	40%	0%	0%	1000s	Ion Torrent	No

Red denotes 14 common mutations shared by all three tumors, while black mutations are unique to the Met2 tumor.

Variant allele frequencies were derived from validation experiments using semiconductor or Sanger sequencing.

Met1, bone marrow metastasis taken at diagnosis; PT, primary tumor taken after cytotoxic therapy; met2, a liver metastasis taken at autopsy.
