# Supplementary material for: Massively Parallel Sequencing Reveals an Accumulation of De Novo Mutations and an Activating Mutation of LPAR1 in a Patient with Metastatic Neuroblastoma
Source: PLoS One. 2013 Oct 16;8(10):e77731. doi: 10.1371/journal.pone.0077731 (PMC3797724; doi:10.1371/journal.pone.0077731)
Supplement: Table S4 — Clinical informaiton of 23 nueroblastoma patients. (PDF) [file pone.0077731.s006.pdf]

Table S4. Clinical informaiton of 23 nueroblastoma patients

Patient ID	Age at Diagnosis (year)	MYCN Status	INSS Staging
NB1006	34	unknown	unknown
NB1007	21	unknown	unknown
NB59	15	Normal copy	4
NB549	15	Amp	2b
NB593	14	Normal copy	4
NB19	13	Normal copy	1
NB42	13	Normal copy	3
NB514	12	Normal copy	4
NB543	11	Normal copy	4
NB113	11	Normal copy	1
NB118	11	Normal copy	4
NB27	11	Amp	4
NB218	10	Amp	4
NB60	10	Normal copy	2
NB541	10	Amp	4
NB575	9	Normal copy	4
NB203	9	Normal copy	3
NB542	8	Normal copy	4
NB250	8	Amp	4
NB532	8	Normal copy	4
NB45	8	Normal copy	4
NB39	8	Normal copy	3
NCI0017	6	unknown	4
