# Supplementary material for: Massively Parallel Sequencing Reveals an Accumulation of De Novo Mutations and an Activating Mutation of LPAR1 in a Patient with Metastatic Neuroblastoma
Source: PLoS One. 2013 Oct 16;8(10):e77731. doi: 10.1371/journal.pone.0077731 (PMC3797724; doi:10.1371/journal.pone.0077731)
Supplement: Table S5 — Gene set enrichment analysis of the change of gene expression profiles in response to LPA (1 hour). (PDF) [file pone.0077731.s007.pdf]

Table S5. Gene set enrichment analysis of the change of gene expression profiles in response to LPA (1 hour).

ID	Size	ES	NES	nominal p	FDR	Description
MILI_PSEUDOPODIA_HAPTOTAXIS_UP	408	0.345835	3.30307	0	0	Transcripts enriched in pseudopodia of NIH/3T3 cells (fibroblast) in response to haptotactic migratory stimulus by fibronectin, FN1 [Gene ID=2335].
FOSTER_INFLAMMATORY_RESPONSE_LPS_DN	316	0.338759	2.87478	0	0.00055	Genes down-regulated by bacterial lipopolysaccharide (LPS) in non-tolerizeable (NT class) macrophages, compared to the ???tolerizeable??? (T class) ones.
BERENJENO_TRANSFORMED_BY_RHOA_UP	435	0.284272	2.78406	0	0.000866667	Genes up-regulated in NIH3T3 cells (fibroblasts) transformed by expression of contitutively active (Q63L) form of RHOA [Gene ID=387] off plasmid vector.
MONNIER_POSTRADIATION_TUMOR_ESCAPE_UP	248	0.34448	2.60202	0	0.00335	The postradiation tumor escape signature: genes up-regulated in tumors from irradiated stroma vs those from non-irradiated stroma.
YAO_TEMPORAL_RESPONSE_TO_PROGESTERONE_CLUSTER_17	150	0.394776	2.39735	0.0006	0.0127667	Genes co-regulated in uterus during a time course response to progesterone [PubChem=5994]: SOM cluster 17.
MARTINEZ_RB1_TARGETS_DN	549	0.21826	2.39302	0.0008	0.0127667	Genes down-regulated in mice with skin specific knockout of RB1 [Gene ID=5925] by Cre-lox.
LEE_LIVER_CANCER_MYC_UP	72	0.523228	2.34275	0.0008	0.0140444	Genes up-regulated in hepatocellular carcinoma (HCC) induced by overexpression of MYC [Gene ID=4609].
GERY_CEBP_TARGETS	92	0.470644	2.3296	0.0011	0.0140444	Genes changed in NIH 3T3 cells (embryonic fibroblast) by expression of one or more of C/EBP proteins: CEBPA, CEBPB, CEBPG, and CEBPD [Gene ID=1050, 1051, 1054, 1052].
MCCLUNG_DELTA_FOSB_TARGETS_8WK	59	0.5598	2.32416	0.0007	0.0140444	Genes up-regulated in the nucleus accumbens (a major reward center in brain) 8 weeks after induction of deltaFosB, a FOSB [Gene ID=2354] splice variant.
MARTINEZ_RB1_AND_TP53_TARGETS_UP	646	0.188525	2.24435	0.0019	0.02278	Genes up-regulated in mice with skin specific double knockout of both RB1 and TP53 [Gene ID=5925, 7157] by Cre-lox.
MARTINEZ_TP53_TARGETS_UP	642	0.187704	2.214	0.0012	0.0254182	Genes up-regulated in mice with skin specific knockout of TP53 [Gene ID=7157].
SEKI_INFLAMMATORY_RESPONSE_LPS_UP	72	0.488564	2.18279	0.0031	0.0268143	Genes up-regulated in hepatic stellar cells after stimulation with bacterial lipopolysacharide (LPS).
MARTORIATI_MDM4_TARGETS_FETAL_LIVER_DN	316	0.257531	2.18036	0.0022	0.0268143	Genes down-regulated in non-apoptotic tissues (fetal liver) after MDM4 [Gene ID=4194] knockout.
MILI_PSEUDOPODIA_CHEMOTAXIS_DN	436	0.221119	2.17015	0.0028	0.0268143	Transcripts depleted in pseudopodia of NIH/3T3 cells (fibroblast) in response to the chemotactic migration stimulus by lysophosphatidic acid (LPA) [PubChem=3988].
STARK_PREFRONTAL_CORTEX_22Q11_DELETION_DN	410	0.223345	2.1461	0.0022	0.0281706	Genes down-regulated in prefrontal cortex (PFC) of mice carrying a hemizygotic microdeletion in the 22q11.2 region.
MARTINEZ_RB1_TARGETS_UP	753	0.168192	2.13672	0.0028	0.0281706	Genes up-regulated in mice with skin specific knockout of RB1 [Gene ID=5925] by Cre-lox.
MARKEY_RB1_ACUTE_LOF_DN	168	-0.333742	-2.13137	0.0023	0.0281706	Genes down-regulated in adult fibroblasts with inactivated RB1 [Gene ID=5925] by Cre-lox: acute loss of function (LOF) of RB1.
MCBRYAN_PUBERTAL_BREAST_6_7WK_UP	242	0.280595	2.10894	0.0035	0.0305167	Genes up-regulated during pubertal mammary gland development between week 6 and 7.
MARTINEZ_TP53_TARGETS_DN	631	0.180177	2.09919	0.0034	0.0307316	Genes down-regulated in mice with skin specific knockout of TP53 [Gene ID=7157].
LIN_NPAS4_TARGETS_UP	103	-0.402862	-2.06983	0.0041	0.03502	Genes up-regulated in neurons after NPAS4 [Gene ID=266743] knockdown by RNAi.
BOYLAN_MULTIPLE_MYELOMA_PCA3_DN	67	-0.469619	-2.04022	0.0059	0.0399286	Top down-regulated genes from principal component 3 which captures variation among different plasma cell tumors arising from overexpression of BCL2L1 and MYC [Gene ID=598, 4609].
LIN_NPAS4_TARGETS_DN	57	0.49535	2.03029	0.0056	0.0404727	Genes down-regulated in neurons after NPAS4 [Gene ID=266743] knockdown by RNAi.
MARTORIATI_MDM4_TARGETS_FETAL_LIVER_UP	90	0.4124	2.01611	0.0066	0.0419913	Genes up-regulated in non-apoptotic tissues (fetal liver) after MDM4 [Gene ID=4194] knockout.
LIAN_LIPA_TARGETS_3M	63	-0.471727	-2.00293	0.0062	0.0434458	Genes up-regulated at 3 months of age in lungs from LIPA [Gene ID=3988] knockout mice, which display pulmonary pathology.
MARTORIATI_MDM4_TARGETS_NEUROEPITHELIUM_DN	70	-0.447576	-1.98158	0.0076	0.04716	Genes down-regulated in apoptotic tissues (neuroepithelium) after MDM4 [Gene ID=4194] knockout.
MARTINEZ_RB1_AND_TP53_TARGETS_DN	623	0.169581	1.97289	0.005	0.0475	Genes down-regulated in mice with skin specific double knockout of both RB1 and TP53 [Gene ID=5925, 7157] by Cre-lox.
MCBRYAN_PUBERTAL_BREAST_6_7WK_DN	74	0.432069	1.95739	0.007	0.0498889	Genes down-regulated during pubertal mammary gland development between week 6 and 7.
BERENJENO_ROCK_SIGNALING_NOT_VIA_RHOA_DN	55	0.475417	1.91631	0.0107	0.0596276	Genes down-regulated in NIH3T3 cells (fibroblasts) after treatment with Y27632 [PubChem=123862], an inhibitor of ROCK proteins; the changes did not depend on expression of constitutively a
OUELLET_OVARIAN_CANCER_INVASIVE_VS_LMP_UP	138	0.327195	1.9107	0.0096	0.0596276	Genes up-regulated in epithelial ovarian cancer (EOC) biopsies: invasive (TOV) vs low malignant potential (LMP) tumors.
BERENJENO_TRANSFORMED_BY_RHOA_DN	385	-0.204043	-1.90072	0.0088	0.0603571	Genes down-regulated in NIH3T3 cells (fibroblasts) transformed by expression of contitutively active (Q63L) form of RHOA [Gene ID=387] off plasmid vector.
RASHI_RESPONSE_TO_IONIZING_RADIATION_3	48	0.491018	1.88337	0.0138	0.0603571	Cluster 3: genes activated by ionizing radiation regardless of ATM [Gene ID=472] status.
BOYLAN_MULTIPLE_MYELOMA_C_DN	44	0.50369	1.87762	0.0133	0.0603571	Genes down-regulated in group C of tumors arising from overexpression of BCL2L1 and MYC [Gene ID=598, 4609] in plasma cells.
MCBRYAN_PUBERTAL_BREAST_5_6WK_DN	159	0.301572	1.87721	0.0133	0.0603571	Genes down-regulated during pubertal mammary gland development between week 5 and 6.
LIAN_LIPA_TARGETS_6M	81	-0.398602	-1.8729	0.013	0.0603571	Genes up-regulated at 6 months of age in lungs from LIPA [Gene ID=3988] knockout mice, which display pulmonary pathology.
MILI_PSEUDOPODIA_HAPTOTAXIS_DN	506	0.176979	1.87171	0.0106	0.0603571	Transcripts depleted from pseudopodia of NIH/3T3 cells (fibroblast) in response to haptotactic migratory stimulus by fibronectin, FN1 [Gene ID=2335].
PAL_PRMT5_TARGETS_UP	200	0.261592	1.80583	0.0174	0.0814083	Genes up-regulated in NIH-3T3 cells (fibroblast) after knockdown of PRMT5 [Gene ID=10419] by RNAi.
YAO_TEMPORAL_RESPONSE_TO_PROGESTERONE_CLUSTER_11	80	-0.383515	-1.79284	0.0191	0.0841243	Genes co-regulated in uterus during a time course response to progesterone [PubChem=5994]: SOM cluster 11.
BOYLAN_MULTIPLE_MYELOMA_PCA3_UP	49	0.45436	1.7563	0.0243	0.0972658	Top up-regulated genes from principal component 3 (PCA3) which captures variation among different plasma cell tumors arising from overexpression of BCL2L1 and MYC [Gene ID=598, 4609].
SWEET_LUNG_CANCER_KRAS_DN	411	0.180378	1.73758	0.0227	0.103451	Genes down-regulated in the mouse lung cancer model with mutated KRAS [Gene ID=3845].
CAIRO_LIVER_DEVELOPMENT_UP	151	0.280686	1.70927	0.0246	0.114743	Genes up-regulated at early fetal liver stage (embryonic days E11.5 - E12.5) compared to the late fetal liver stage (embryonic days E14.5 - E16.5).
MORI_SMALL_PRE_BII_LYMPHOCYTE_DN	58	0.405514	1.67806	0.0302	0.128898	Down-regulated genes in the B lymphocyte developmental signature, based on expression profiling of lymphomas from the Emu-myc transgenic mice: the Small Pre-BII stage.
BYSTRYKH_HEMATOPOIESIS_STEM_CELL_QTL_CIS	123	0.299122	1.66913	0.0329	0.13096	Transcripts in hematopoietic stem cells (HSC) which are cis-regulated (i.e., modulated by a QTL (quantitative trait locus) in close proximity to the gene).
LEE_LIVER_CANCER_E2F1_DN	53	0.412429	1.64307	0.0403	0.140977	Genes down-regulated in hepatocellular carcinoma (HCC) induced by overexpression of E2F1 [Gene ID=1869].
ZHANG_BREAST_CANCER_PROGENITORS_UP	279	0.20488	1.64026	0.0368	0.140977	Genes changed in cancer stem cells isolated from mammary tumors compared to the non-tumorigenic cells.
MCBRYAN_PUBERTAL_BREAST_4_5WK_UP	227	0.223216	1.63375	0.0347	0.140977	Genes up-regulated during pubertal mammary gland development between week 4 and 5.
BOYLAN_MULTIPLE_MYELOMA_D_DN	61	-0.387285	-1.62294	0.0395	0.140977	Genes down-regulated in group D of tumors arising from overexpression of BCL2L1 and MYC [Gene ID=598, 4609] in plasma cells.
HESS_TARGETS_OF_HOXA9_AND_MEIS1_DN	87	0.333637	1.62106	0.0422	0.140977	Genes down-regulated in hematopoietic precursor cells conditionally expressing HOXA9 and MEIS1 [Gene ID=3205, 4211].
RIZ_ERYTHROID_DIFFERENTIATION	79	0.349456	1.62102	0.0405	0.140977	Selected gradually up-regulated genes in the TLX1 [Gene ID=3195] Tet On iEBHX15-4 cells (pro-erythroblasts).
TSENG_IRS1_TARGETS_DN	113	0.299955	1.61059	0.0399	0.144686	Down-regulated in brown preadipocytes with IRS1 [Gene ID=3667] knockout vs wild type controls; the knockouts have severe defects in adipocyte differentiation.
LEE_LIVER_CANCER_MYC_DN	40	0.441904	1.59409	0.0531	0.152252	Genes down-regulated in hepatocellular carcinoma (HCC) induced by overexpression of MYC [Gene ID=4609].
GOLDRATH_ANTIGEN_RESPONSE	277	0.200134	1.58859	0.0423	0.152504	Genes up-regulated at the peak of an antigen response of naive CD8+ [Gene ID=925, 926] T-cells.
MORI_LARGE_PRE_BII_LYMPHOCYTE_UP	51	0.404802	1.584	0.0545	0.152504	Up-regulated genes in the B lymphocyte developmental signature, based on expression profiling of lymphomas from the Emu-myc transgenic mice: the Large Pre-BII stage.
BYSTRYKH_HEMATOPOIESIS_STEM_CELL_QTL_TRANS	866	0.114258	1.55375	0.063	0.169031	Transcripts in hematopoietic stem cells (HSC) which are trans-regulated (i.e., modulated by a QTL (quantitative trait locus) not in a close proximity to the gene).
LEE_TARGETS_OF_PTCH1_AND_SUFU_DN	60	-0.370849	-1.55063	0.0555	0.169031	Genes down-regulated in medulloblastoma tumors from animals with inactivating mutations of one copy of PTCH1 or SUFU [Gene ID=5727, 51684] in conjunction with TP53 [Gene ID=7157] lo
YAO_TEMPORAL_RESPONSE_TO_PROGESTERONE_CLUSTER_7	56	0.369744	1.50484	0.0671	0.200805	Genes co-regulated in uterus during a time course response to progesterone [PubChem=5994]: SOM cluster 7.
KHETCHOUMIAN_TRIM24_TARGETS_UP	72	0.334964	1.49716	0.0622	0.20357	Retinoic acid-responsive genes up-regulated in hepatocellular carcinoma (HCC) samples of TRIM24 [Gene ID=8805] knockout mice.
MARTORIATI_MDM4_TARGETS_NEUROEPITHELIUM_UP	89	0.304265	1.47662	0.0713	0.217839	Genes up-regulated in apoptotic tissues (neuroepithelium) after MDM4 [Gene ID=4194] knockout.
YAUCH_HEDGEHOG_SIGNALING_PARACRINE_UP	136	-0.249426	-1.44018	0.0839	0.247343	Genes up-regulated in mouse stroma of pancreatic adenocarcinoma zenografts after treatment with HhAntag, a hedgehog (Hh) pathway inhibitor.
MCCLUNG_DELTA_FOSB_TARGETS_2WK	62	0.341105	1.43364	0.0896	0.247343	Genes up-regulated in the nucleus accumbens (a major reward center in brain) 2 weeks after induction of deltaFosB, a FOSB [Gene ID=2354] splice variant.
MILI_PSEUDOPODIA_CHEMOTAXIS_UP	67	0.329586	1.43016	0.0853	0.247343	Transcripts enriched in pseudopodia of NIH/3T3 cells (fibroblast) in response to the chemotactic migration stimulus by lysophosphatidic acid (LPA) [PubChem=3988].
MARSON_FOXP3_TARGETS_UP	63	0.338205	1.42861	0.0923	0.247343	Genes up-regulated by FOXP3 [Gene ID=50943] in both ex vivo and hybridoma cells.
BREDEMEYER_RAG_SIGNALING_NOT_VIA_ATM_DN	66	0.327202	1.41969	0.0891	0.249744	Genes down-regulated in pre B lymphocyte after induction of physiological DNA double-strand breaks (DSB) by RAG2 [Gene ID=5897]; the changes are independent of ATM [Gene ID=472] signa
KENNY_CTNNB1_TARGETS_UP	34	-0.41452	-1.41804	0.1257	0.249744	Genes up-regulated in HC11 cells (mammary epithelium) by expression of constantly active CTNNB1 [Gene ID=1499].
RIZ_ERYTHROID_DIFFERENTIATION_CCNE1	41	0.37936	1.39073	0.1265	0.274734	Selected gradually up-regulated genes whose expression profile follows that of CCNE1 [Gene ID=898] in the TLX1 [Gene ID=3195] Tet On iEBHX15-4 cells (pro-erythroblasts).
MORI_PRE_BI_LYMPHOCYTE_DN	57	0.336552	1.3792	0.1082	0.283214	Down-regulated genes in the B lymphocyte developmental signature, based on expression profiling of lymphomas from the Emu-myc transgenic mice: the Pre-BI stage.
MARKEY_RB1_CHRONIC_LOF_UP	112	0.25694	1.37433	0.1074	0.284009	Genes up-regulated in MEF cells (embryonic fibroblasts) isolated from RB1 [Gene ID=5925] knockout mice: chronic loss of function (LOF) of RB1.
HESS_TARGETS_OF_HOXA9_AND_MEIS1_UP	93	0.275581	1.36597	0.1116	0.284009	Genes up-regulated in hematopoietic precursor cells conditionally expressing HOXA9 and MEIS1 [Gene ID=3205, 4211].

BOYLAN_MULTIPLE_MYELOMA_C_D_UP	113	0.253123	1.36399	0.1097	0.284009	Genes up-regulated both in group C and D of tumors arising from overexpression of BCL2L1 and MYC [Gene ID=598, 4609] in plasma cells.
KENNY_CTNNB1_TARGETS_DN	66	0.316476	1.36334	0.1145	0.284009	Genes down-regulated in HC11 cells (mammary epithelium) by expression of constantly active CTNNB1 [Gene ID=1499].
YAO_TEMPORAL_RESPONSE_TO_PROGESTERONE_CLUSTER_16	87	0.280654	1.34921	0.1184	0.295971	Genes co-regulated in uterus during a time course response to progesterone [PubChem=5994]: SOM cluster 16.
MATSUDA_NATURAL_KILLER_DIFFERENTIATION	437	0.13525	1.33026	0.1435	0.309842	Genes changed between developmental stages of Valpha14i natural killer T lymphocyte cells (NKT).
LEE_LIVER_CANCER_MYC_TGFA_DN	50	0.341258	1.3283	0.1423	0.309842	Genes down-regulated in hepatocellular carcinoma (HCC) tissue of MYC and TGFA [Gene ID=4609, 7039] double transgenic mice.
IVANOVA_HEMATOPOIESIS_STEM_CELL_AND_PROGENITOR	214	-0.18713	-1.32671	0.1352	0.309842	Genes in the expression cluster 'HSC and Progenitors Shared': up-regulated in hematopoietic stem cells (HSC) and progenitors from adult bone marrow and fetal liver.
YAO_TEMPORAL_RESPONSE_TO_PROGESTERONE_CLUSTER_13	141	0.223764	1.31674	0.1373	0.317741	Genes co-regulated in uterus during a time course response to progesterone [PubChem=5994]: SOM cluster 13.
BYSTRYKH_HEMATOPOIESIS_STEM_CELL_AND_BRAIN_QTL_CIS	57	0.315831	1.29331	0.1566	0.336686	Genes associated with the same cis-regulatory QTL (quantitative trait loci) in both brain and hematopoietic stem cells (HSC).
MARKEY_RB1_CHRONIC_LOF_DN	101	-0.250937	-1.28582	0.1456	0.336686	Genes down-regulated in MEF cells (embryonic fibroblasts) isolated from RB1 [Gene ID=5925] knockout mice: chronic loss of function (LOF) of RB1.
MCCLUNG_CREB1_TARGETS_UP	100	0.251703	1.2837	0.1497	0.336686	Genes up-regulated in the nucleus accumbens (a major reward center in the brain) 8 weeks after induction of CREB1 [Gene ID=1385] expression in a transgenic Tet-Off system.
YU_MYC_TARGETS_DN	52	0.32386	1.2818	0.1689	0.336686	Genes down-regulated in B cell lymphoma tumors expressing an activated form of MYC [Gene ID=4609].
STEARMAN_LUNG_CANCER_EARLY_VS_LATE_UP	96	0.254396	1.28146	0.1534	0.336686	Genes classifying non-tumor lung tissues by age after incution of lung cancer by urethane injection [PubChem=5641]: early (24-26 weeks) vs late (46 weeks).
IVANOVA_HEMATOPOIESIS_EARLY_PROGENITOR	66	0.296689	1.28117	0.1548	0.336686	Genes in the expression cluster 'Early Progenitors Shared': up-regulated in hematopoietic progenitors from adult bone marrow and from fetal liver.
GOLDRATH_IMMUNE_MEMORY	46	-0.33719	-1.27724	0.1783	0.337526	'Memory genes' expressed uniquely in CD8+ [Gene ID=925] memory T lymphocytes (compared with effector or na??ve cells)
TSENG_IRS1_TARGETS_UP	119	0.228828	1.25724	0.1677	0.359641	Up-regulated in brown preadipocytes with IRS1 [Gene ID=3667] knockout vs wild type controls; the knockouts have severe defects in adipocyte differentiation.
IWANAGA_CARCINOGENESIS_BY_KRAS_PTEN_UP	174	0.193652	1.25115	0.1855	0.363404	Cluster 1: genes up-regulated in lung tissue samples from mice with oncogenic form of KRAS [Gene ID=3845] and inactivated PTEN [Gene ID=5728].
IWANAGA_CARCINOGENESIS_BY_KRAS_DN	111	-0.231141	-1.23901	0.1786	0.375613	Cluster 4: genes down-regulated in lung tissue samples from mice with tumor-bearing genotypes (activated KRAS [Gene ID=3845] alone or together with inactivated PTEN [Gene ID=5728]).
TSENG_ADIPOGENIC_POTENTIAL_DN	45	0.326443	1.23551	0.2037	0.376071	Genes showing decreasing expression in brown preadipocytes with increasing ability of the cells to differentiate.
RASHI_RESPONSE_TO_IONIZING_RADIATION_2	127	0.216835	1.22602	0.1884	0.384034	Cluster 2: late ATM [Gene ID=472] dependent genes induced by ionizing radiation treatment.
IWANAGA_CARCINOGENESIS_BY_KRAS_UP	156	0.197754	1.22347	0.1958	0.384034	Cluster 3: genes up-regulated in lung tissue samples from mice with tumor-bearing genotypes (activated KRAS [Gene ID=3845] alone or together with inactivated PTEN [Gene ID=5728]).
KUMAR_TARGETS_OF_MLL_AF9_FUSION	347	0.137665	1.21998	0.2198	0.384461	Myeloid leukemia model in mice with germ-line MLL-AF9 fusion knock-in [Gene ID=4297, 4300]: genes changed in comparison among the leukemic, preleukemic and wild-type animals.
IVANOVA_HEMATOPOIESIS_MATURE_CELL	119	-0.219585	-1.21259	0.1957	0.390645	Genes in the expression cluster 'MBC Shared': up-regulated in mature blood cell populations from adult bone marrow and fetal liver.
SWEET_LUNG_CANCER_KRAS_UP	430	0.123779	1.20776	0.2327	0.393257	Genes up-regulated in the mouse lung cancer model with mutated KRAS [Gene ID=3845].
YAUCH_HEDGEHOG_SIGNALING_PARACRINE_DN	265	0.153241	1.20353	0.2235	0.395013	Genes down-regulated in mouse stroma of pancreatic adenocarcinoma zenografts after treatment with HhAntag, a hedgehog (Hh) pathway inhibitor.
LEE_LIVER_CANCER_E2F1_UP	60	0.285096	1.1884	0.2283	0.412442	Genes up-regulated in hepatocellular carcinoma (HCC) induced by overexpression of E2F1 [Gene ID=1869].
YAO_TEMPORAL_RESPONSE_TO_PROGESTERONE_CLUSTER_10	52	-0.298681	-1.18588	0.2296	0.412442	Genes co-regulated in uterus during a time course response to progesterone [PubChem=5994]: SOM cluster 11.
RASHI_RESPONSE_TO_IONIZING_RADIATION_5	135	0.202594	1.17372	0.2364	0.423682	Cluster 5: early responding genes activated in ATM [Gene ID=472] deficient but not in the wild type tissues.
YAO_HOXA10_TARGETS_VIA_PROGESTERONE_UP	74	0.259348	1.17249	0.2308	0.423682	Genes up-regulated in the uteri of ovariectomized mice 6 h after progesterone [PubChem=5994] injection: HOXA10 [Gene ID=3206] knockout vs wild type animals.
CHESLER_BRAIN_QTL_CIS	69	0.263252	1.15774	0.2459	0.437104	Best cis-regulated quantitative trait loci (QTLs) in the mouse genome which modulate transcription in brain tissue.
PASQUALUCCI_LYMPHOMA_BY_GC_STAGE_UP	181	0.174955	1.15536	0.2645	0.437104	Genes up-regulated in post-GC, BCL6 [Gene ID=604] dependent B cell non-Hodgkin's lymphoma (B-NHL) vs MYC [Gene ID=4609] driven pre-GC lymphoma; GC = germinal center.
CADWELL_ATG16L1_TARGETS_UP	63	0.27119	1.15352	0.2532	0.437104	Genes up-regulated in Paneth cell (part of intestiinal epithelium) of mice with hypomorphic (reduced function) form of ATG16L1 [Gene ID=55054].
UEDA_CENTRAL_CLOCK	74	0.253857	1.15228	0.2497	0.437104	Molecular timetable composed of 96 time-indicating genes (103 probes) in the central (suprachiasmatic nucleus (SCN)) clock.
YAO_TEMPORAL_RESPONSE_TO_PROGESTERONE_CLUSTER_12	79	-0.246085	-1.14737	0.2474	0.44025	Genes co-regulated in uterus during a time course response to progesterone [PubChem=5994]: SOM cluster 12.
RAMALHO_STEMNESS_UP	162	-0.177929	-1.11908	0.2942	0.479876	Genes enriched in embryonic, neural and hematopoietic stem cells.
IVANOVA_HEMATOPOIESIS_STEM_CELL	71	0.250632	1.11694	0.2835	0.479876	Genes in the expression cluster 'HSC Shared': up-regulated in hematopoietic stem cells (HSC) from adult bone marrow and fetal liver.
BOYLAN_MULTIPLE_MYELOMA_D_UP	59	-0.26867	-1.10942	0.2976	0.484743	Genes up-regulated in group D of tumors arising from overexpression of BCL2L1 and MYC [Gene ID=598, 4609] in plasma cells.
ICHIBA_GRAFT_VERSUS_HOST_DISEASE_D7_UP	95	-0.221518	-1.10839	0.2933	0.484743	Hepatic graft versus host disease (GVHD), day 7: up-regulated in allogeneic vs syngeneic bone marrow transplant.
MORI_MATURE_B_LYMPHOCYTE_DN	60	-0.263334	-1.09736	0.3133	0.496095	Down-regulated genes in the B lymphocyte developmental signature, based on expression profiling of lymphomas from the Emu-myc transgenic mice: the mature B
LEE_LIVER_CANCER_DENA_UP	65	0.255595	1.09615	0.3096	0.496095	Genes up-regulated in hepatocellular carcinoma (HCC) induced by diethylnitrosamine (DENA) [PubChem=5921].
LEE_LIVER_CANCER_MYC_TGFA_UP	76	0.239846	1.09	0.3146	0.501074	Genes up-regulated in hepatocellular carcinoma (HCC) tissue of MYC and TGFA [Gene ID=4609, 7039] double transgenic mice.
YAO_TEMPORAL_RESPONSE_TO_PROGESTERONE_CLUSTER_1	61	0.25946	1.0861	0.3232	0.501074	Genes co-regulated in uterus during a time course response to progesterone [PubChem=5994]: SOM cluster 1.
LEE_LIVER_CANCER_MYC_E2F1_DN	45	0.289615	1.08487	0.3361	0.501074	Genes down-regulated in hepatocellular carcinoma (HCC) from MYC and E2F1 [Gene ID=4609, 1869] double transgenic mice.
GOLDRATH_HOMEOSTATIC_PROLIFERATION	137	0.186099	1.07996	0.3275	0.504819	Up-regulated in CD8+ [Gene ID=925] T lymphocytes undergoing homeostatic proliferation (HP) versus the naive cells; these genes are not up-regulated versus effector or memory cell populatic
MONNIER_POSTRADIATION_TUMOR_ESCAPE_DN	226	-0.148524	-1.07553	0.3523	0.507716	The postradiation tumor escape signature: genes down-regulated in tumors from irradiated stroma vs those from non-irradiated stroma.
MORI_IMMATURE_B_LYMPHOCYTE_DN	44	0.287845	1.07054	0.348	0.509691	Down-regulated genes in the B lymphocyte developmental signature based on expression profiling of lymphomas from the Emu-myc transgenic mice: the immature B stage.
BOYLAN_MULTIPLE_MYELOMA_PCA1_UP	114	-0.198232	-1.06891	0.3368	0.509691	Top up-regulated genes from principal component 1 (PCA1) which captures variation between normal plasma cells and tumors arising from aberrant expression of BCL2L1 and MYC [Gene ID=5
YAO_TEMPORAL_RESPONSE_TO_PROGESTERONE_CLUSTER_0	66	0.24582	1.05992	0.349	0.519298	Genes co-regulated in uterus during a time course response to progesterone [PubChem=5994]: SOM cluster 0.
MORI_EMU_MYC_LYMPHOMA_BY_ONSET_TIME_UP	101	-0.206174	-1.05671	0.3538	0.519298	Genes correlated with the early tumor onset in the Emu-myc transgenic mouse lymphoma model.
ZHANG_BREAST_CANCER_PROGENITORS_DN	104	0.2029	1.05536	0.3543	0.519298	Genes changed in cancer stem cells isolated from mammary tumors compared to the non-tumorigenic cells.
HOFFMANN_LARGE_TO_SMALL_PRE_BII_LYMPHOCYTE_UP	69	0.233966	1.0327	0.3922	0.55398	Genes up-regulated during differentiation from large pre-BII to small pre-BII lymphocyte.
IWANAGA_CARCINOGENESIS_BY_KRAS_PTEN_DN	314	0.120777	1.02495	0.4249	0.563061	Cluster 2: genes down-regulated in lung tissue samples from mice with oncogenic form of KRAS [Gene ID=3845] and inactivated PTEN [Gene ID=5728].
MORI_LARGE_PRE_BII_LYMPHOCYTE_DN	45	-0.27013	-1.01844	0.4001	0.570045	Down-regulated genes in the B lymphocyte developmental signature, based on expression profiling of lymphomas from the Emu-myc transgenic mice: the Large Pre-BII stage.
STEARMAN_LUNG_CANCER_EARLY_VS_LATE_DN	60	-0.240849	-0.999222	0.425	0.595008	Genes classifying non-tumor lung tissues by age after incution of lung cancer by urethane injection [PubChem=5641]: early (24-26 weeks) vs late (46 weeks).
MCBRYAN_PUBERTAL_BREAST_3_4WK_UP	178	0.152644	0.997043	0.4472	0.595008	Genes up-regulated during pubertal mammary gland development between weeks 3 and 4.
MARSON_FOXP3_TARGETS_DN	59	0.241603	0.996566	0.4296	0.595008	Genes down-regulated by FOXP3 [Gene ID=50943] in both ex vivo and hybridoma cells.
MORI_IMMATURE_B_LYMPHOCYTE_UP	40	-0.269061	-0.972164	0.4595	0.635145	Up-regulated genes in the B lymphocyte developmental signature based on expression profiling of lymphomas from the Emu-myc transgenic mice: the immature B stage.
RASHI_RESPONSE_TO_IONIZING_RADIATION_4	53	-0.242648	-0.967726	0.4725	0.638312	Cluster 4: genes repressed by ionizing radiation regardless of ATM [Gene ID=472] status.
RAMALHO_STEMNESS_DN	75	-0.209476	-0.958295	0.4877	0.64605	Genes depleted in embryonic, neural and hematopoietic stem cells.
LIANG_HEMATOPOIESIS_STEM_CELL_NUMBER_LARGE_VS_TINY_UP	46	-0.253092	-0.958073	0.4804	0.64605	Genes up-regulated in LSK cells (bone marrow) as a function of a QTL for the size of hematopoietic stem cell (HSC) population: comparison of congenic B.D. chr3 (BD, large HSC size) vs parental
CAIRO_LIVER_DEVELOPMENT_DN	165	-0.150807	-0.955328	0.5031	0.64605	Genes down-regulated at early fetal liver stage (embryonic days E11.5 - E12.5) compared to the late fetal liver stage (embryonic days E14.5 - E16.5).
IVANOVA_HEMATOPOIESIS_LATE_PROGENITOR	114	0.175829	0.950241	0.504	0.648253	Genes in the expression cluster 'Late Progenitors Shared': up-regulated in hematopoietic late progenitor cells from adult bone marrow and fetal liver.
BOYLAN_MULTIPLE_MYELOMA_C_D_DN	217	0.132516	0.948611	0.5209	0.648253	Genes down-regulated both in group C and D of tumors arising from overexpression of BCL2L1 and MYC [Gene ID=598, 4609] in plasma cells.
COATES_MACROPHAGE_M1_VS_M2_DN	88	0.192484	0.931944	0.5281	0.674004	Down-regulated genes distinguishing between M1 (pro-inflammatory) and M2 (anti-inflammatory) macrophage subtypes.
LE_EGR2_TARGETS_UP	63	0.217021	0.921873	0.5404	0.687417	Genes up-regulated in P14 nerves of transgenic mice having hypomorhic (reduced function) allele of EGR2 [Gene ID=1959].
YAO_TEMPORAL_RESPONSE_TO_PROGESTERONE_CLUSTER_14	74	0.201353	0.909888	0.5594	0.70432	Genes co-regulated in uterus during a time course response to progesterone [PubChem=5994]: SOM cluster 14.
BYSTRYKH_HEMATOPOIESIS_STEM_CELL_AND_BRAIN_QTL_TRANS	139	-0.153781	-0.902829	0.5845	0.711257	Genes trans-regulated by the same QTL (quantitative trait loci) in brain and hematopoietic stem cells (HSC).
LE_EGR2_TARGETS_DN	106	0.17155	0.896144	0.5847	0.711257	Genes down-regulated in P14 nerves of transgenic mice having hypomorhic (reduced function) allele of EGR2 [Gene ID=1959].
YAO_TEMPORAL_RESPONSE_TO_PROGESTERONE_CLUSTER_6	55	0.221371	0.893733	0.5759	0.711257	Genes co-regulated in uterus during a time course response to progesterone [PubChem=5994]: SOM cluster 6.
ICHIBA_GRAFT_VERSUS_HOST_DISEASE_35D_UP	116	-0.163995	-0.892281	0.5982	0.711257	Hepatic graft versus host disease (GVHD), day 35: genes up-regulated in allogeneic vs syngeneic bone marrow transplant.
PASQUALUCCI_LYMPHOMA_BY_GC_STAGE_DN	133	0.155218	0.891769	0.5949	0.711257	Genes down-regulated in post-GC, BCL6 [Gene ID=604] dependent B cell non-Hodgkin's lymphoma (B-NHL) vs MYC [Gene ID=4609] driven pre-GC lymphoma; GC = germinal center.

UEDA_PERIFERAL_CLOCK	114	-0.163174	-0.883369	0.6068	0.721214	Molecular timetable composed of 162 time-indicating genes (182 probes) in the peripheral (liver) clock.
YAO_TEMPORAL_RESPONSE_TO_PROGESTERONE_CLUSTER_9	73	0.194918	0.875746	0.6156	0.729867	Genes co-regulated in uterus during a time course response to progesterone [PubChem=5994]: SOM cluster 9.
STARK_PREFRONTAL_CORTEX_22Q11_DELETION_UP	179	-0.131371	-0.860733	0.6434	0.751516	Genes up-regulated in prefrontal cortex (PFC) of mice carrying a hemizygotic microdeletion in the 22q11.2 region.
COATES_MACROPHAGE_M1_VS_M2_UP	60	0.199616	0.836038	0.6761	0.790211	Up-regulated genes distinguishing between M1 (pro-inflammatory) and M2 (anti-inflammatory) macrophage subtypes.
IVANOVA_HEMATOPOIESIS_STEM_CELL_LONG_TERM	77	0.180623	0.828642	0.6927	0.797521	Genes in the expression cluster 'LT-HSC Shared': up-regulated in long term hematopoietic stem cells (LT-HSC) from adult bone marrow and fetal liver.
MORI_SMALL_PRE_BII_LYMPHOCYTE_UP	59	0.197147	0.820263	0.6937	0.806434	Up-regulated genes in the B lymphocyte developmental signature, based on expression profiling of lymphomas from the Emu-myc transgenic mice: the Small Pre-BII stage.
RIZ_ERYTHROID_DIFFERENTIATION_12HR	50	0.209326	0.816341	0.6846	0.807459	Selected genes down-regulated in the TLX1 [Gene ID=3195] Tet On iEBHX15-4 cells (pro-erythroblasts) at 12 h time point.
HOFFMANN_LARGE_TO_SMALL_PRE_BII_LYMPHOCYTE_DN	38	0.222692	0.795801	0.702	0.836477	Genes down-regulated during differentiation from large pre-BII to small pre-BII lymphocyte.
LEE_LIVER_CANCER_MYC_E2F1_UP	47	0.206356	0.789613	0.7206	0.839803	Genes up-regulated in hepatocellular carcinoma (HCC) from MYC and E2F1 [Gene ID=4609, 1869] double transgenic mice.
MCBRYAN_PUBERTAL_BREAST_4_5WK_DN	222	0.109535	0.786713	0.7396	0.839803	Genes down-regulated during pubertal mammary gland development between week 4 and 5.
MORI_MATURE_B_LYMPHOCYTE_UP	63	0.184406	0.782439	0.747	0.841022	Up-regulated genes in the B lymphocyte developmental signature, based on expression profiling of lymphomas from the Emu-myc transgenic mice: the mature B
CADWELL_ATG16L1_TARGETS_DN	56	0.189822	0.774351	0.758	0.847995	Genes down-regulated in Paneth cell (part of intestiinal epithelium) of mice with hypomorphic (reduced function) form of ATG16L1 [Gene ID=55054].
MCBRYAN_PUBERTAL_BREAST_5_6WK_UP	91	-0.15524	-0.760884	0.7879	0.86123	Genes up-regulated during pubertal mammary gland development between week 5 and 6.
LEE_LIVER_CANCER_CIPROFIBRATE_UP	54	-0.189058	-0.758315	0.7795	0.86123	Genes up-regulated in hepatocellular carcinoma (HCC) induced by ciprofibrate [PubChem=2763].
KUNINGER_IGF1_VS_PDGFB_TARGETS_UP	54	-0.183849	-0.739182	0.8076	0.883461	Genes up-regulated in C2AS12 cells (myoblast) by IGF1 [Gene ID=3479] vs PDGFB [Gene ID=5155].
ICHIBA_GRAFT_VERSUS_HOST_DISEASE_D7_DN	40	-0.202125	-0.730896	0.7989	0.889267	Hepatic graft versus host disease (GVHD), day 7: down-regulated in allogeneic vs syngeneic bone marrow transplant.
HOFFMANN_PRE_BI_TO_LARGE_PRE_BII_LYMPHOCYTE_DN	46	0.191259	0.724919	0.8129	0.889267	Genes down-regulated during differentiation from pre-BI to large pre-BII lymphocyte.
LEE_LIVER_CANCER_ACOX1_DN	51	0.183046	0.719488	0.8291	0.889267	Genes down-regulated in hepatocellular carcinoma of ACOX1 [Gene ID=51] knockout mice.
LEE_TARGETS_OF_PTCH1_AND_SUFU_UP	102	0.138253	0.716049	0.8626	0.889267	Genes up-regulated in medulloblastoma tumors from animals with inactivating mutations of one copy of PTCH1 or SUFU [Gene ID=5727, 51684] in conjunction with TP53 [Gene ID=7157] loss.
MARKEY_RB1_ACUTE_LOF_UP	218	0.0993849	0.713806	0.8268	0.889267	Genes up-regulated in adult fibroblasts with inactivated RB1 [Gene ID=5925] by Cre-lox: acute loss of function (LOF) of RB1.
ICHIBA_GRAFT_VERSUS_HOST_DISEASE_35D_DN	53	-0.177402	-0.706922	0.8438	0.892387	Hepatic graft versus host disease (GVHD), day 35: genes down-regulated in allogeneic vs syngeneic bone marrow transplant.
RIZ_ERYTHROID_DIFFERENTIATION_6HR	40	-0.192008	-0.697742	0.8413	0.898057	Selected genes down-regulated in the TLX1 [Gene ID=3195] Tet On iEBHX15-4 cells (pro-erythroblasts) at 6 h time point.
LEE_LIVER_CANCER_ACOX1_UP	46	0.175696	0.666565	0.8833	0.927271	Genes up-regulated in hepatocellular carcinoma of ACOX1 [Gene ID=51] knockout mice.
MCMURRAY_TP53_HRAS_COOPERATION_RESPONSE_DN	49	-0.165934	-0.638833	0.9136	0.948316	Down-regulated 'cooperation response genes': responded synergystically to the combination of mutant TP53 [Gene ID=7157] and HRAS [Gene ID=3265] in YAMC cells (colon).
MCCLUNG_CREB1_TARGETS_DN	52	-0.159586	-0.632023	0.9312	0.948556	Genes down-regulated in the nucleus accumbens (a major reward center in the brain) 8 weeks after induction of CREB1 [Gene ID=1385] expression in a transgenic Tet-Off system.
FOSTER_INFLAMMATORY_RESPONSE_LPS_UP	157	0.097062	0.599953	0.9499	0.967147	Genes up-regulated by bacterial lipopolysaccharide (LPS) in non-tolerizeable (NT class) macrophages, compared to the ???tolerizeable??? (T class) ones.
LEE_LIVER_CANCER_DENA_DN	54	0.132275	0.533114	0.9866	0.994344	Genes down-regulated in hepatocellular carcinoma (HCC) induced by diethylnitrosamine (DENA) [PubChem=5921].
LEE_LIVER_CANCER_CIPROFIBRATE_DN	49	0.132276	0.516141	0.9906	0.994344	Genes down-regulated in hepatocellular carcinoma (HCC) induced by ciprofibrate [PubChem=2763].
RASHI_RESPONSE_TO_IONIZING_RADIATION_6	70	0.109925	0.485842	0.997	0.994552	Cluster 6: late responding genes activated in ATM [Gene ID=472] deficient but not in the wild type tissues
