# Supplementary material for: Massively Parallel Sequencing Reveals an Accumulation of De Novo Mutations and an Activating Mutation of LPAR1 in a Patient with Metastatic Neuroblastoma
Source: PLoS One. 2013 Oct 16;8(10):e77731. doi: 10.1371/journal.pone.0077731 (PMC3797724; doi:10.1371/journal.pone.0077731)
Supplement: Table S6 — Gene set enrichment analysis of the change of gene expression profiles in response to LPA (2 hours). (PDF) [file pone.0077731.s008.pdf]

Table S6. Gene set enrichment analysis of the change of gene expression profiles in response to LPA (2 hours).

ID	Size	ES	NES	nominal p	FDR	Description
MILI_PSEUDOPODIA_HAPTOTAXIS_UP	408	0.296115	3.01573	0	0.0005	Transcripts enriched in pseudopodia of NIH/3T3 cells (fibroblast) in response to haptotactic migratory stimulus by fibronectin, FN1 [Gene ID=2335].
MONNIER_POSTRADIATION_TUMOR_ESCAPE_UP	248	0.343568	2.77056	0.0001	0.00265	The postradiation tumor escape signature: genes up-regulated in tumors from irradiated stroma vs those from non-irradiated stroma.
SWEET_LUNG_CANCER_KRAS_UP	430	0.233234	2.42633	0.0001	0.0275667	Genes up-regulated in the mouse lung cancer model with mutated KRAS [Gene ID=3845].
ICHIBA_GRAFT_VERSUS_HOST_DISEASE_35D_UP	116	-0.38387	-2.22866	0.0029	0.0822	Hepatic graft versus host disease (GVHD), day 35: genes up-regulated in allogeneic vs syngeneic bone marrow transplant.
YAO_TEMPORAL_RESPONSE_TO_PROGESTERONE_CLUSTER_14	74	0.443047	2.12916	0.0043	0.119533	Genes co-regulated in uterus during a time course response to progesterone [PubChem=5994]: SOM cluster 14.
BYSTRYKH_HEMATOPOIESIS_STEM_CELL_QTL_TRANS	866	0.145075	2.09829	0.004	0.119533	Transcripts in hematopoietic stem cells (HSC) which are trans-regulated (i.e., modulated by a QTL (quantitative trait locus) not in a close proximity to the gene).
FOSTER_INFLAMMATORY_RESPONSE_LPS_DN	316	0.222761	2.00806	0.0053	0.16045	Genes down-regulated by bacterial lipopolysaccharide (LPS) in non-tolerizeable (NT class) macrophages, compared to the ???tolerizeable??? (T class) ones.
YAO_TEMPORAL_RESPONSE_TO_PROGESTERONE_CLUSTER_11	80	0.39436	1.94847	0.0101	0.16045	Genes co-regulated in uterus during a time course response to progesterone [PubChem=5994]: SOM cluster 11.
BERENJENO_TRANSFORMED_BY_RHOA_UP	435	0.184184	1.93854	0.0083	0.16045	Genes up-regulated in NIH3T3 cells (fibroblasts) transformed by expression of contitutively active (Q63L) form of RHOA [Gene ID=387] off plasmid vector.
MILI_PSEUDOPODIA_HAPTOTAXIS_DN	506	0.17242	1.93298	0.0075	0.16045	Transcripts depleted from pseudopodia of NIH/3T3 cells (fibroblast) in response to haptotactic migratory stimulus by fibronectin, FN1 [Gene ID=2335].
MARTINEZ_RB1_TARGETS_UP	753	0.140523	1.91148	0.0083	0.16045	Genes up-regulated in mice with skin specific knockout of RB1 [Gene ID=5925] by Cre-lox.
BERENJENO_ROCK_SIGNALING_NOT_VIA_RHOA_DN	55	0.440236	1.89055	0.013	0.16045	Genes down-regulated in NIH3T3 cells (fibroblasts) after treatment with Y27632 [PubChem=123862], an inhibitor of ROCK proteins; the changes did not depend on expression of constitutively active (Q63L) form of RHOA [Gene ID=387].
MCCLUNG_DELTA_FOSB_TARGETS_8WK	59	0.430979	1.8902	0.0154	0.16045	Genes up-regulated in the nucleus accumbens (a major reward center in brain) 8 weeks after induction of deltaFosB, a FOSB [Gene ID=2354] splice variant.
YAO_TEMPORAL_RESPONSE_TO_PROGESTERONE_CLUSTER_16	87	-0.3675	-1.88131	0.0133	0.16045	Genes co-regulated in uterus during a time course response to progesterone [PubChem=5994]: SOM cluster 16.
PASQUALUCCI_LYMPHOMA_BY_GC_STAGE_UP	181	0.26373	1.8545	0.0123	0.16968	Genes up-regulated in post-GC, BCL6 [Gene ID=604] dependent B cell non-Hodgkin's lymphoma (B-NHL) vs MYC [Gene ID=4609] driven pre-GC lymphoma; GC = germinal center.
BERENJENO_TRANSFORMED_BY_RHOA_DN	385	-0.1791	-1.77603	0.0176	0.224841	Genes down-regulated in NIH3T3 cells (fibroblasts) transformed by expression of contitutively active (Q63L) form of RHOA [Gene ID=387] off plasmid vector.
MCMURRAY_TP53_HRAS_COOPERATION_RESPONSE_DN	49	-0.43292	-1.76496	0.0232	0.224841	Down-regulated 'cooperation response genes': responded synergystically to the combination of mutant TP53 [Gene ID=7157] and HRAS [Gene ID=3265] in YAMC cells (colon).
KENNY_CTNNB1_TARGETS_DN	66	0.378515	1.73786	0.0292	0.231579	Genes down-regulated in HC11 cells (mammary epithelium) by expression of constantly active CTNNB1 [Gene ID=1499].
IWANAGA_CARCINOGENESIS_BY_KRAS_UP	156	0.264002	1.73266	0.0225	0.231579	Cluster 3: genes up-regulated in lung tissue samples from mice with tumor-bearing genotypes (activated KRAS [Gene ID=3845] alone or together with inactivated PTEN [Gene ID=5728]).
SEKI_INFLAMMATORY_RESPONSE_LPS_UP	72	0.359582	1.70975	0.0325	0.242062	Genes up-regulated in hepatic stellar cells after stimulation with bacterial lipopolysacharide (LPS).
OUELLET_OVARIAN_CANCER_INVASIVE_VS_LMP_UP	138	0.271968	1.69945	0.0288	0.242062	Genes up-regulated in epithelial ovarian cancer (EOC) biopsies: invasive (TOV) vs low malignant potential (LMP) tumors.
MCBRYAN_PUBERTAL_BREAST_6_7WK_DN	74	0.34882	1.67759	0.0357	0.253345	Genes down-regulated during pubertal mammary gland development between week 6 and 7.
SWEET_LUNG_CANCER_KRAS_DN	411	0.162041	1.65534	0.033	0.26613	Genes down-regulated in the mouse lung cancer model with mutated KRAS [Gene ID=3845].
ZHANG_BREAST_CANCER_PROGENITORS_UP	279	0.192776	1.63715	0.0363	0.271865	Genes changed in cancer stem cells isolated from mammary tumors compared to the non-tumorigenic cells.
MILI_PSEUDOPODIA_CHEMOTAXIS_DN	436	0.154857	1.62725	0.0361	0.271865	Transcripts depleted in pseudopodia of NIH/3T3 cells (fibroblast) in response to the chemotactic migration stimulus by lysophosphatidic acid (LPA) [PubChem=3988].
MORI_SMALL_PRE_BII_LYMPHOCYTE_DN	58	0.372423	1.62072	0.0434	0.271865	Down-regulated genes in the B lymphocyte developmental signature, based on expression profiling of lymphomas from the Emu-myc transgenic mice: the Small Pre-BII stage.
RAMALHO_STEMNESS_DN	75	0.329106	1.59169	0.0452	0.295156	Genes depleted in embryonic, neural and hematopoietic stem cells.
MARTINEZ_TP53_TARGETS_DN	631	0.121102	1.51625	0.0666	0.337939	Genes down-regulated in mice with skin specific knockout of TP53 [Gene ID=7157].
BOYLAN_MULTIPLE_MYELOMA_D_DN	61	-0.34153	-1.51611	0.0658	0.337939	Genes down-regulated in group D of tumors arising from overexpression of BCL2L1 and MYC [Gene ID=598, 4609] in plasma cells.
HOFFMANN_LARGE_TO_SMALL_PRE_BII_LYMPHOCYTE_UP	69	-0.32381	-1.51127	0.0618	0.337939	Genes up-regulated during differentiation from large pre-BII to small pre-BII lymphocyte.
MARTINEZ_RB1_AND_TP53_TARGETS_DN	623	0.121844	1.50836	0.0669	0.337939	Genes down-regulated in mice with skin specific double knockout of both RB1 and TP53 [Gene ID=5925, 7157] by Cre-lox.
LE_EGR2_TARGETS_DN	106	0.271236	1.50499	0.0675	0.337939	Genes down-regulated in P14 nerves of transgenic mice having hypomorhic (reduced function) allele of EGR2 [Gene ID=1959].
RIZ_ERYTHROID_DIFFERENTIATION	79	-0.30562	-1.50319	0.0696	0.337939	Selected gradually up-regulated genes in the TLX1 [Gene ID=3195] Tet On iEBHX15-4 cells (pro-erythroblasts).
KUNINGER_IGF1_VS_PDGFB_TARGETS_UP	54	0.352854	1.49582	0.0734	0.337939	Genes up-regulated in C2AS12 cells (myoblast) by IGF1 [Gene ID=3479] vs PDGFB [Gene ID=5155].
RIZ_ERYTHROID_DIFFERENTIATION_6HR	40	0.393513	1.49164	0.0893	0.337939	Selected genes down-regulated in the TLX1 [Gene ID=3195] Tet On iEBHX15-4 cells (pro-erythroblasts) at 6 h time point.
MARTORIATI_MDM4_TARGETS_FETAL_LIVER_UP	90	0.286886	1.48689	0.0692	0.337939	Genes up-regulated in non-apoptotic tissues (fetal liver) after MDM4 [Gene ID=4194] knockout.
GOLDRATH_ANTIGEN_RESPONSE	277	0.17219	1.46436	0.078	0.342515	Genes up-regulated at the peak of an antigen response of naive CD8+ [Gene ID=925, 926] T-cells.
LEE_LIVER_CANCER_MYC_DN	40	0.383528	1.46223	0.0982	0.342515	Genes down-regulated in hepatocellular carcinoma (HCC) induced by overexpression of MYC [Gene ID=4609].
MCCLUNG_CREB1_TARGETS_UP	100	-0.27091	-1.46083	0.081	0.342515	Genes up-regulated in the nucleus accumbens (a major reward center in the brain) 8 weeks after induction of CREB1 [Gene ID=1385] expression in a transgenic Tet-Off system.
MCBRYAN_PUBERTAL_BREAST_5_6WK_UP	91	0.278513	1.45568	0.0782	0.342515	Genes up-regulated during pubertal mammary gland development between week 5 and 6.
ZHANG_BREAST_CANCER_PROGENITORS_DN	104	0.262556	1.45097	0.0796	0.342515	Genes changed in cancer stem cells isolated from mammary tumors compared to the non-tumorigenic cells.
MARTORIATI_MDM4_TARGETS_NEUROEPITHELIUM_UP	89	0.277834	1.43645	0.0837	0.350725	Genes up-regulated in apoptotic tissues (neuroepithelium) after MDM4 [Gene ID=4194] knockout.
LEE_LIVER_CANCER_MYC_UP	72	0.30115	1.43086	0.0896	0.350725	Genes up-regulated in hepatocellular carcinoma (HCC) induced by overexpression of MYC [Gene ID=4609].
STARK_PREFRONTAL_CORTEX_22Q11_DELETION_DN	410	-0.13959	-1.42734	0.0964	0.350725	Genes down-regulated in prefrontal cortex (PFC) of mice carrying a hemizygotic microdeletion in the 22q11.2 region.
YAO_TEMPORAL_RESPONSE_TO_PROGESTERONE_CLUSTER_13	141	-0.22569	-1.41504	0.0921	0.359831	Genes co-regulated in uterus during a time course response to progesterone [PubChem=5994]: SOM cluster 13.
CADWELL_ATG16L1_TARGETS_UP	63	0.311445	1.40658	0.0942	0.363365	Genes up-regulated in Paneth cell (part of intestiinal epithelium) of mice with hypomorphic (reduced function) form of ATG16L1 [Gene ID=55054].
MATSUDA_NATURAL_KILLER_DIFFERENTIATION	437	0.132308	1.39746	0.1069	0.363365	Genes changed between developmental stages of Valpha14i natural killer T lymphocyte cells (NKT).
STEARMAN_LUNG_CANCER_EARLY_VS_LATE_UP	96	0.26103	1.39604	0.1038	0.363365	Genes classifying non-tumor lung tissues by age after incution of lung cancer by urethane injection [PubChem=5641]: early (24-26 weeks) vs late (46 weeks).
MCBRYAN_PUBERTAL_BREAST_4_5WK_DN	222	0.180093	1.38222	0.1076	0.368314	Genes down-regulated during pubertal mammary gland development between week 4 and 5.
RAMALHO_STEMNESS_UP	162	0.207446	1.38123	0.1061	0.368314	Genes enriched in embryonic, neural and hematopoietic stem cells.
IWANAGA_CARCINOGENESIS_BY_KRAS_DN	111	0.242292	1.37695	0.1044	0.368314	Cluster 4: genes down-regulated in lung tissue samples from mice with tumor-bearing genotypes (activated KRAS [Gene ID=3845] alone or together with inactivated PTEN [Gene ID=5728]).
YAO_TEMPORAL_RESPONSE_TO_PROGESTERONE_CLUSTER_17	150	-0.21179	-1.36571	0.1136	0.377685	Genes co-regulated in uterus during a time course response to progesterone [PubChem=5994]: SOM cluster 17.
LIN_NPAS4_TARGETS_UP	103	0.238698	1.31508	0.1342	0.448391	Genes up-regulated in neurons after NPAS4 [Gene ID=266743] knockdown by RNAi.
MARKEY_RB1_CHRONIC_LOF_UP	112	-0.22914	-1.31146	0.1397	0.448391	Genes up-regulated in MEF cells (embryonic fibroblasts) isolated from RB1 [Gene ID=5925] knockout mice: chronic loss of function (LOF) of RB1.
RASHI_RESPONSE_TO_IONIZING_RADIATION_2	127	0.213597	1.28658	0.1554	0.476646	Cluster 2: late ATM [Gene ID=472] dependent genes induced by ionizing radiation treatment.
BOYLAN_MULTIPLE_MYELOMA_PCA1_UP	114	0.222687	1.28518	0.1491	0.476646	Top up-regulated genes from principal component 1 (PCA1) which captures variation between normal plasma cells and tumors arising from aberrant expression of BCL2L1 and MYC [Gene ID=598, 4609].
YAO_TEMPORAL_RESPONSE_TO_PROGESTERONE_CLUSTER_10	52	-0.30664	-1.28116	0.1716	0.476646	Genes co-regulated in uterus during a time course response to progesterone [PubChem=5994]: SOM cluster 11.
MCCLUNG_DELTA_FOSB_TARGETS_2WK	62	0.282547	1.26883	0.1692	0.4836	Genes up-regulated in the nucleus accumbens (a major reward center in brain) 2 weeks after induction of deltaFosB, a FOSB [Gene ID=2354] splice variant.
ICHIBA_GRAFT_VERSUS_HOST_DISEASE_D7_DN	40	-0.33365	-1.26733	0.1957	0.4836	Hepatic graft versus host disease (GVHD), day 7: down-regulated in allogeneic vs syngeneic bone marrow transplant.
MCBRYAN_PUBERTAL_BREAST_6_7WK_UP	242	0.158243	1.26178	0.1793	0.4836	Genes up-regulated during pubertal mammary gland development between week 6 and 7.
STARK_PREFRONTAL_CORTEX_22Q11_DELETION_UP	179	-0.17983	-1.25697	0.1767	0.4836	Genes up-regulated in prefrontal cortex (PFC) of mice carrying a hemizygotic microdeletion in the 22q11.2 region.
MILI_PSEUDOPODIA_CHEMOTAXIS_UP	67	0.272417	1.25501	0.1731	0.4836	Transcripts enriched in pseudopodia of NIH/3T3 cells (fibroblast) in response to the chemotactic migration stimulus by lysophosphatidic acid (LPA) [PubChem=3988].
MCCLUNG_CREB1_TARGETS_DN	52	0.297896	1.24723	0.1935	0.489849	Genes down-regulated in the nucleus accumbens (a major reward center in the brain) 8 weeks after induction of CREB1 [Gene ID=1385] expression in a transgenic Tet-Off system.
LEE_TARGETS_OF_PTCH1_AND_SUFU_DN	60	-0.27904	-1.23377	0.1951	0.506861	Genes down-regulated in medulloblastoma tumors from animals with inactivating mutations of one copy of PTCH1 or SUFU [Gene ID=5727, 51684] in conjunction with TP53 [Gene ID=7157] loss.
YAO_TEMPORAL_RESPONSE_TO_PROGESTERONE_CLUSTER_7	56	0.284296	1.22002	0.2098	0.525043	Genes co-regulated in uterus during a time course response to progesterone [PubChem=5994]: SOM cluster 7.
RIZ_ERYTHROID_DIFFERENTIATION_CCNE1	41	-0.31331	-1.20805	0.2331	0.537906	Selected gradually up-regulated genes whose expression profile follows that of CCNE1 [Gene ID=898] in the TLX1 [Gene ID=3195] Tet On iEBHX15-4 cells (pro-erythroblasts).
MORI_LARGE_PRE_BII_LYMPHOCYTE_UP	51	-0.28915	-1.20504	0.2229	0.537906	Up-regulated genes in the B lymphocyte developmental signature, based on expression profiling of lymphomas from the Emu-myc transgenic mice: the Large Pre-BII stage.
KUMAR_TARGETS_OF_MLL_AF9_FUSION	347	0.125449	1.18913	0.2433	0.561218	Myeloid leukemia model in mice with germ-line MLL-AF9 fusion knock-in [Gene ID=4297, 4300]: genes changed in comparison among the leukemic, preleukemic and wild-type animals.
MARTINEZ_RB1_TARGETS_DN	549	-0.0996	-1.16809	0.2652	0.584472	Genes down-regulated in mice with skin specific knockout of RB1 [Gene ID=5925] by Cre-lox.
FOSTER_INFLAMMATORY_RESPONSE_LPS_UP	157	0.176784	1.16628	0.2451	0.584472	Genes up-regulated by bacterial lipopolysaccharide (LPS) in non-tolerizeable (NT class) macrophages, compared to the ???tolerizeable??? (T class) ones.
LEE_TARGETS_OF_PTCH1_AND_SUFU_UP	102	0.214337	1.16577	0.2371	0.584472	Genes up-regulated in medulloblastoma tumors from animals with inactivating mutations of one copy of PTCH1 or SUFU [Gene ID=5727, 51684] in conjunction with TP53 [Gene ID=7157] loss.
IVANOVA_HEMATOPOIESIS_STEM_CELL_LONG_TERM	77	-0.23349	-1.13384	0.2667	0.63457	Genes in the expression cluster 'LT-HSC Shared': up-regulated in long term hematopoietic stem cells (LT-HSC) from adult bone marrow and fetal liver.
YAO_TEMPORAL_RESPONSE_TO_PROGESTERONE_CLUSTER_9	73	-0.23718	-1.13087	0.2705	0.63457	Genes co-regulated in uterus during a time course response to progesterone [PubChem=5994]: SOM cluster 9.
IVANOVA_HEMATOPOIESIS_MATURE_CELL	119	-0.19156	-1.11912	0.2795	0.63457	Genes in the expression cluster 'MBC Shared': up-regulated in mature blood cell populations from adult bone marrow and fetal liver.
BYSTRYKH_HEMATOPOIESIS_STEM_CELL_QTL_CIS	123	0.187883	1.11684	0.2831	0.63457	Transcripts in hematopoietic stem cells (HSC) which are cis-regulated (i.e., modulated by a QTL (quantitative trait locus) in close proximity to the gene).
TSENG_IRS1_TARGETS_DN	113	0.192891	1.10469	0.2941	0.63457	Down-regulated in brown preadipocytes with IRS1 [Gene ID=3667] knockout vs wild type controls; the knockouts have severe defects in adipocyte differentiation.
LIN_NPAS4_TARGETS_DN	57	0.255678	1.10411	0.3092	0.63457	Genes down-regulated in neurons after NPAS4 [Gene ID=266743] knockdown by RNAi.
LEE_LIVER_CANCER_E2F1_DN	53	0.261733	1.10226	0.3179	0.63457	Genes down-regulated in hepatocellular carcinoma (HCC) induced by overexpression of E2F1 [Gene ID=1869].
COATES_MACROPHAGE_M1_VS_M2_DN	88	0.213299	1.10136	0.3021	0.63457	Down-regulated genes distinguishing between M1 (pro-inflammatory) and M2 (anti-inflammatory) macrophage subtypes.
RASHI_RESPONSE_TO_IONIZING_RADIATION_5	135	0.178205	1.09862	0.3064	0.63457	Cluster 5: early responding genes activated in ATM [Gene ID=472] deficient but not in the wild type tissues.
BOYLAN_MULTIPLE_MYELOMA_C_D_DN	217	0.143952	1.09829	0.332	0.63457	Genes down-regulated both in group C and D of tumors arising from overexpression of BCL2L1 and MYC [Gene ID=598, 4609] in plasma cells.
IVANOVA_HEMATOPOIESIS_STEM_CELL_AND_PROGENITOR	214	0.145211	1.09709	0.3286	0.63457	Genes in the expression cluster 'HSC and Progenitors Shared': up-regulated in hematopoietic stem cells (HSC) and progenitors from adult bone marrow and fetal liver.
MARSON_FOXP3_TARGETS_DN	59	0.250317	1.09675	0.3152	0.63457	Genes down-regulated by FOXP3 [Gene ID=50943] in both ex vivo and hybridoma cells.
BOYLAN_MULTIPLE_MYELOMA_C_DN	44	0.274718	1.08225	0.336	0.653045	Genes down-regulated in group C of tumors arising from overexpression of BCL2L1 and MYC [Gene ID=598, 4609] in plasma cells.
LE_EGR2_TARGETS_UP	63	0.24034	1.08092	0.3311	0.653045	Genes up-regulated in P14 nerves of transgenic mice having hypomorhic (reduced function) allele of EGR2 [Gene ID=1959].
PASQUALUCCI_LYMPHOMA_BY_GC_STAGE_DN	133	0.174918	1.07423	0.3312	0.660017	Genes down-regulated in post-GC, BCL6 [Gene ID=604] dependent B cell non-Hodgkin's lymphoma (B-NHL) vs MYC [Gene ID=4609] driven pre-GC lymphoma; GC = germinal center.
UEDA_PERIFERAL_CLOCK	114	-0.18115	-1.04637	0.3599	0.714516	Molecular timetable composed of 162 time-indicating genes (182 probes) in the peripheral (liver) clock.
MARTINEZ_RB1_AND_TP53_TARGETS_UP	646	0.083034	1.04103	0.3954	0.718642	Genes up-regulated in mice with skin specific double knockout of both RB1 and TP53 [Gene ID=5925, 7157] by Cre-lox.
GOLDRATH_HOMEOSTATIC_PROLIFERATION	137	0.16652	1.03202	0.3872	0.720593	Up-regulated in CD8+ [Gene ID=925] T lymphocytes undergoing homeostatic proliferation (HP) versus the naive cells; these genes are not up-regulated versus effector or memory cell population.
HESS_TARGETS_OF_HOXA9_AND_MEIS1_UP	93	0.195879	1.02955	0.3821	0.720593	Genes up-regulated in hematopoietic precursor cells conditionally expressing HOXA9 and MEIS1 [Gene ID=3205, 4211].
LIAN_LIPA_TARGETS_3M	63	0.230048	1.02949	0.3879	0.720593	Genes up-regulated at 3 months of age in lungs from LIPA [Gene ID=3988] knockout mice, which display pulmonary pathology.
YU_MYC_TARGETS_DN	52	0.243401	1.02075	0.4077	0.732312	Genes down-regulated in B cell lymphoma tumors expressing an activated form of MYC [Gene ID=4609].
LEE_LIVER_CANCER_DENA_UP	65	0.219746	1.00413	0.4258	0.743237	Genes up-regulated in hepatocellular carcinoma (HCC) induced by diethylnitrosamine (DENA) [PubChem=5921].
MORI_EMU_MYC_LYMPHOMA_BY_ONSET_TIME_UP	101	0.183995	1.00364	0.4142	0.743237	Genes correlated with the early tumor onset in the Emu-myc transgenic mouse lymphoma model.
IVANOVA_HEMATOPOIESIS_STEM_CELL	71	0.214222	1.00349	0.423	0.743237	Genes in the expression cluster 'HSC Shared': up-regulated in hematopoietic stem cells (HSC) from adult bone marrow and fetal liver.
CAIRO_LIVER_DEVELOPMENT_UP	151	0.155001	1.00198	0.4268	0.743237	Genes up-regulated at early fetal liver stage (embryonic days E11.5 - E12.5) compared to the late fetal liver stage (embryonic days E14.5 - E16.5).
IWANAGA_CARCINOGENESIS_BY_KRAS_PTEN_DN	314	0.110256	0.998326	0.4479	0.743897	Cluster 2: genes down-regulated in lung tissue samples from mice with oncogenic form of KRAS [Gene ID=3845] and inactivated PTEN [Gene ID=5728].
MCBRYAN_PUBERTAL_BREAST_3_4WK_UP	178	-0.14282	-0.993534	0.4473	0.746881	Genes up-regulated during pubertal mammary gland development between weeks 3 and 4.
GOLDRATH_IMMUNE_MEMORY	46	0.244443	0.977589	0.4536	0.770125	'Memory genes' expressed uniquely in CD8+ [Gene ID=925] memory T lymphocytes (compared with effector or na??ve cells)
LIAN_LIPA_TARGETS_6M	81	0.19638	0.976318	0.4587	0.770125	Genes up-regulated at 6 months of age in lungs from LIPA [Gene ID=3988] knockout mice, which display pulmonary pathology.
MCBRYAN_PUBERTAL_BREAST_5_6WK_DN	159	-0.14553	-0.969005	0.4776	0.778699	Genes down-regulated during pubertal mammary gland development between week 5 and 6.
GERY_CEBP_TARGETS	92	0.18209	0.960757	0.4866	0.787587	Genes changed in NIH 3T3 cells (embryonic fibroblast) by expression of one or more of C/EBP proteins: CEBPA, CEBPB, CEBPG, and CEBPD [Gene ID=1050, 1051, 1054, 1052].

COATES_MACROPHAGE_M1_VS_M2_UP	60	-0.21674	-0.957892	0.4858	0.787587	Up-regulated genes distinguishing between M1 (pro-inflammatory) and M2 (anti-inflammatory) macrophage subtypes.
KENNY_CTNNB1_TARGETS_UP	34	0.262754	0.946836	0.4801	0.787587	Genes up-regulated in HC11 cells (mammary epithelium) by expression of constantly active CTNNB1 [Gene ID=1499].
IVANOVA_HEMATOPOIESIS_EARLY_PROGENITOR	66	0.205208	0.939467	0.5088	0.787587	Genes in the expression cluster 'Early Progenitors Shared': up-regulated in hematopoietic progenitors from adult bone marrow and from fetal liver.
BYSTRYKH_HEMATOPOIESIS_STEM_CELL_AND_BRAIN_QTL_CIS	57	0.21745	0.937778	0.5128	0.787587	Genes associated with the same cis-regulatory QTL (quantitative trait loci) in both brain and hematopoietic stem cells (HSC).
MARSON_FOXP3_TARGETS_UP	63	0.208046	0.936079	0.5175	0.787587	Genes up-regulated by FOXP3 [Gene ID=50943] in both ex vivo and hybridoma cells.
MORI_PRE_BI_LYMPHOCYTE_DN	57	0.214417	0.931381	0.5253	0.787587	Down-regulated genes in the B lymphocyte developmental signature, based on expression profiling of lymphomas from the Emu-myc transgenic mice: the Pre-BI stage.
LEE_LIVER_CANCER_MYC_E2F1_DN	45	0.234531	0.930558	0.5169	0.787587	Genes down-regulated in hepatocellular carcinoma (HCC) from MYC and E2F1 [Gene ID=4609, 1869] double transgenic mice.
STEARMAN_LUNG_CANCER_EARLY_VS_LATE_DN	60	0.211352	0.923577	0.5304	0.787587	Genes classifying non-tumor lung tissues by age after incution of lung cancer by urethane injection [PubChem=5641]: early (24-26 weeks) vs late (46 weeks).
MARTINEZ_TP53_TARGETS_UP	642	0.073342	0.923007	0.5439	0.787587	Genes up-regulated in mice with skin specific knockout of TP53 [Gene ID=7157].
BOYLAN_MULTIPLE_MYELOMA_PCA3_UP	49	-0.22589	-0.922796	0.5301	0.787587	Top up-regulated genes from principal component 3 (PCA3) which captures variation among different plasma cell tumors arising from overexpression of BCL2L1 and MYC [Gene ID=598, 4609].
MARTORIATI_MDM4_TARGETS_FETAL_LIVER_DN	316	0.101566	0.918584	0.5569	0.787587	Genes down-regulated in non-apoptotic tissues (fetal liver) after MDM4 [Gene ID=4194] knockout.
BOYLAN_MULTIPLE_MYELOMA_D_UP	59	-0.20845	-0.915446	0.5427	0.787587	Genes up-regulated in group D of tumors arising from overexpression of BCL2L1 and MYC [Gene ID=598, 4609] in plasma cells.
YAUCH_HEDGEHOG_SIGNALING_PARACRINE_UP	136	0.14648	0.911781	0.5611	0.787587	Genes up-regulated in mouse stroma of pancreatic adenocarcinoma zenografts after treatment with HhAntag, a hedgehog (Hh) pathway inhibitor.
MORI_SMALL_PRE_BII_LYMPHOCYTE_UP	59	-0.20884	-0.911725	0.5532	0.787587	Up-regulated genes in the B lymphocyte developmental signature, based on expression profiling of lymphomas from the Emu-myc transgenic mice: the Small Pre-BII stage.
YAUCH_HEDGEHOG_SIGNALING_PARACRINE_DN	265	0.109069	0.908442	0.5723	0.787587	Genes down-regulated in mouse stroma of pancreatic adenocarcinoma zenografts after treatment with HhAntag, a hedgehog (Hh) pathway inhibitor.
ICHIBA_GRAFT_VERSUS_HOST_DISEASE_D7_UP	95	0.170693	0.90611	0.5653	0.787587	Hepatic graft versus host disease (GVHD), day 7: up-regulated in allogeneic vs syngeneic bone marrow transplant.
IVANOVA_HEMATOPOIESIS_LATE_PROGENITOR	114	0.157008	0.904187	0.5734	0.787587	Genes in the expression cluster 'Late Progenitors Shared': up-regulated in hematopoietic late progenitor cells from adult bone marrow and fetal liver.
MCBRYAN_PUBERTAL_BREAST_4_5WK_UP	227	0.116129	0.901994	0.584	0.787587	Genes up-regulated during pubertal mammary gland development between week 4 and 5.
LEE_LIVER_CANCER_ACOX1_UP	46	0.221864	0.890408	0.5696	0.800478	Genes up-regulated in hepatocellular carcinoma of ACOX1 [Gene ID=51] knockout mice.
CHESLER_BRAIN_QTL_CIS	69	0.189935	0.883797	0.5874	0.800478	Best cis-regulated quantitative trait loci (QTLs) in the mouse genome which modulate transcription in brain tissue.
YAO_TEMPORAL_RESPONSE_TO_PROGESTERONE_CLUSTER_1	61	-0.19936	-0.881343	0.5918	0.800478	Genes co-regulated in uterus during a time course response to progesterone [PubChem=5994]: SOM cluster 1.
TSENG_IRS1_TARGETS_UP	119	0.15087	0.880564	0.6127	0.800478	Up-regulated in brown preadipocytes with IRS1 [Gene ID=3667] knockout vs wild type controls; the knockouts have severe defects in adipocyte differentiation.
HESS_TARGETS_OF_HOXA9_AND_MEIS1_DN	87	0.171765	0.879315	0.6106	0.800478	Genes down-regulated in hematopoietic precursor cells conditionally expressing HOXA9 and MEIS1 [Gene ID=3205, 4211].
MORI_LARGE_PRE_BII_LYMPHOCYTE_DN	45	0.2193	0.870651	0.5968	0.811048	Down-regulated genes in the B lymphocyte developmental signature, based on expression profiling of lymphomas from the Emu-myc transgenic mice: the Large Pre-BII stage.
PAL_PRMT5_TARGETS_UP	200	-0.1171	-0.85898	0.6379	0.826388	Genes up-regulated in NIH-3T3 cells (fibroblast) after knockdown of PRMT5 [Gene ID=10419] by RNAi.
MARKEY_RB1_ACUTE_LOF_UP	218	0.112636	0.856171	0.6474	0.826388	Genes up-regulated in adult fibroblasts with inactivated RB1 [Gene ID=5925] by Cre-lox: acute loss of function (LOF) of RB1.
LIANG_HEMATOPOIESIS_STEM_CELL_NUMBER_LARGE_VS_TINY_UP	46	0.212547	0.849508	0.6292	0.827286	Genes up-regulated in LSK cells (bone marrow) as a function of a QTL for the size of hematopoietic stem cell (HSC) population: comparison of congenic B.D. chr3 (BD, large HSC size) vs parental B6 strain (tiny HSC size).
RASHI_RESPONSE_TO_IONIZING_RADIATION_3	48	0.207165	0.847798	0.632	0.827286	Cluster 3: genes activated by ionizing radiation regardless of ATM [Gene ID=472] status.
YAO_TEMPORAL_RESPONSE_TO_PROGESTERONE_CLUSTER_0	66	-0.18492	-0.843646	0.6616	0.827286	Genes co-regulated in uterus during a time course response to progesterone [PubChem=5994]: SOM cluster 0.
MORI_IMMATURE_B_LYMPHOCYTE_UP	40	0.219309	0.842404	0.6372	0.827286	Up-regulated genes in the B lymphocyte developmental signature based on expression profiling of lymphomas from the Emu-myc transgenic mice: the immature B stage.
HOFFMANN_PRE_BI_TO_LARGE_PRE_BII_LYMPHOCYTE_DN	46	0.206803	0.828817	0.6582	0.846368	Genes down-regulated during differentiation from pre-BI to large pre-BII lymphocyte.
MARKEY_RB1_ACUTE_LOF_DN	168	-0.12158	-0.822695	0.6906	0.851246	Genes down-regulated in adult fibroblasts with inactivated RB1 [Gene ID=5925] by Cre-lox: acute loss of function (LOF) of RB1.
BOYLAN_MULTIPLE_MYELOMA_C_D_UP	113	0.141043	0.810753	0.7252	0.860925	Genes up-regulated both in group C and D of tumors arising from overexpression of BCL2L1 and MYC [Gene ID=598, 4609] in plasma cells.
YAO_TEMPORAL_RESPONSE_TO_PROGESTERONE_CLUSTER_12	79	0.163756	0.8086	0.7199	0.860925	Genes co-regulated in uterus during a time course response to progesterone [PubChem=5994]: SOM cluster 12.
RASHI_RESPONSE_TO_IONIZING_RADIATION_4	53	-0.19098	-0.806743	0.6998	0.860925	Cluster 4: genes repressed by ionizing radiation regardless of ATM [Gene ID=472] status.
TSENG_ADIPOGENIC_POTENTIAL_DN	45	0.200527	0.80161	0.6962	0.863728	Genes showing decreasing expression in brown preadipocytes with increasing ability of the cells to differentiate.
YAO_HOXA10_TARGETS_VIA_PROGESTERONE_UP	74	0.161704	0.781399	0.7606	0.892004	Genes up-regulated in the uteri of ovariectomized mice 6 h after progesterone [PubChem=5994] injection: HOXA10 [Gene ID=3206] knockout vs wild type animals.
LEE_LIVER_CANCER_MYC_E2F1_UP	47	-0.19077	-0.770826	0.7456	0.896487	Genes up-regulated in hepatocellular carcinoma (HCC) from MYC and E2F1 [Gene ID=4609, 1869] double transgenic mice.
LEE_LIVER_CANCER_MYC_TGFA_DN	50	0.185616	0.764475	0.7567	0.896487	Genes down-regulated in hepatocellular carcinoma (HCC) tissue of MYC and TGFA [Gene ID=4609, 7039] double transgenic mice.
YAO_TEMPORAL_RESPONSE_TO_PROGESTERONE_CLUSTER_6	55	0.178436	0.763676	0.7699	0.896487	Genes co-regulated in uterus during a time course response to progesterone [PubChem=5994]: SOM cluster 6.
LEE_LIVER_CANCER_CIPROFIBRATE_UP	54	-0.18011	-0.763223	0.7611	0.896487	Genes up-regulated in hepatocellular carcinoma (HCC) induced by ciprofibrate [PubChem=2763].
ICHIBA_GRAFT_VERSUS_HOST_DISEASE_35D_DN	53	-0.1763	-0.745499	0.7873	0.90962	Hepatic graft versus host disease (GVHD), day 35: genes down-regulated in allogeneic vs syngeneic bone marrow transplant.
HOFFMANN_LARGE_TO_SMALL_PRE_BII_LYMPHOCYTE_DN	38	0.19863	0.743046	0.7732	0.90962	Genes down-regulated during differentiation from large pre-BII to small pre-BII lymphocyte.
LEE_LIVER_CANCER_MYC_TGFA_UP	76	0.152793	0.742244	0.817	0.90962	Genes up-regulated in hepatocellular carcinoma (HCC) tissue of MYC and TGFA [Gene ID=4609, 7039] double transgenic mice.
CADWELL_ATG16L1_TARGETS_DN	56	-0.17073	-0.735945	0.8057	0.90962	Genes down-regulated in Paneth cell (part of intestiinal epithelium) of mice with hypomorphic (reduced function) form of ATG16L1 [Gene ID=55054].
MONNIER_POSTRADIATION_TUMOR_ESCAPE_DN	226	-0.09468	-0.733817	0.8055	0.90962	The postradiation tumor escape signature: genes down-regulated in tumors from irradiated stroma vs those from non-irradiated stroma.
MARTORIATI_MDM4_TARGETS_NEUROEPITHELIUM_DN	70	-0.15481	-0.727634	0.8285	0.90989	Genes down-regulated in apoptotic tissues (neuroepithelium) after MDM4 [Gene ID=4194] knockout.
LEE_LIVER_CANCER_E2F1_UP	60	0.164545	0.725058	0.8177	0.90989	Genes up-regulated in hepatocellular carcinoma (HCC) induced by overexpression of E2F1 [Gene ID=1869].
MORI_IMMATURE_B_LYMPHOCYTE_DN	44	0.181019	0.714206	0.8185	0.917726	Down-regulated genes in the B lymphocyte developmental signature based on expression profiling of lymphomas from the Emu-myc transgenic mice: the immature B stage.
BREDEMEYER_RAG_SIGNALING_NOT_VIA_ATM_DN	66	-0.15331	-0.702577	0.8551	0.917726	Genes down-regulated in pre B lymphocyte after induction of physiological DNA double-strand breaks (DSB) by RAG2 [Gene ID=5897]; the changes are independent of ATM [Gene ID=472] signaling.
KHETCHOUMIAN_TRIM24_TARGETS_UP	72	-0.14845	-0.702286	0.8584	0.917726	Retinoic acid-responsive genes up-regulated in hepatocellular carcinoma (HCC) samples of TRIM24 [Gene ID=8805] knockout mice.
UEDA_CENTRAL_CLOCK	74	0.145538	0.698146	0.8725	0.917726	Molecular timetable composed of 96 time-indicating genes (103 probes) in the central (suprachiasmatic nucleus (SCN)) clock.
BOYLAN_MULTIPLE_MYELOMA_PCA3_DN	67	-0.15019	-0.692176	0.8733	0.917726	Top down-regulated genes from principal component 3 which captures variation among different plasma cell tumors arising from overexpression of BCL2L1 and MYC [Gene ID=598, 4609].
RIZ_ERYTHROID_DIFFERENTIATION_12HR	50	-0.16556	-0.682651	0.8612	0.917726	Selected genes down-regulated in the TLX1 [Gene ID=3195] Tet On iEBHX15-4 cells (pro-erythroblasts) at 12 h time point.
CAIRO_LIVER_DEVELOPMENT_DN	165	-0.1009	-0.680595	0.8753	0.917726	Genes down-regulated at early fetal liver stage (embryonic days E11.5 - E12.5) compared to the late fetal liver stage (embryonic days E14.5 - E16.5).
MORI_MATURE_B_LYMPHOCYTE_UP	63	0.150014	0.67712	0.8876	0.917726	Up-regulated genes in the B lymphocyte developmental signature, based on expression profiling of lymphomas from the Emu-myc transgenic mice: the mature B
MORI_MATURE_B_LYMPHOCYTE_DN	60	-0.15255	-0.67592	0.8802	0.917726	Down-regulated genes in the B lymphocyte developmental signature, based on expression profiling of lymphomas from the Emu-myc transgenic mice: the mature B
LEE_LIVER_CANCER_ACOX1_DN	51	-0.15503	-0.64571	0.9047	0.943097	Genes down-regulated in hepatocellular carcinoma of ACOX1 [Gene ID=51] knockout mice.
MARKEY_RB1_CHRONIC_LOF_DN	101	0.113955	0.623252	0.9441	0.954948	Genes down-regulated in MEF cells (embryonic fibroblasts) isolated from RB1 [Gene ID=5925] knockout mice: chronic loss of function (LOF) of RB1.
RASHI_RESPONSE_TO_IONIZING_RADIATION_6	70	0.131308	0.619058	0.9444	0.954948	Cluster 6: late responding genes activated in ATM [Gene ID=472] deficient but not in the wild type tissues.
BYSTRYKH_HEMATOPOIESIS_STEM_CELL_AND_BRAIN_QTL_TRANS	139	-0.09692	-0.606859	0.9438	0.958625	Genes trans-regulated by the same QTL (quantitative trait loci) in brain and hematopoietic stem cells (HSC).
IWANAGA_CARCINOGENESIS_BY_KRAS_PTEN_UP	174	-0.085	-0.587063	0.9567	0.966518	Cluster 1: genes up-regulated in lung tissue samples from mice with oncogenic form of KRAS [Gene ID=3845] and inactivated PTEN [Gene ID=5728].
LEE_LIVER_CANCER_DENA_DN	54	-0.116	-0.493779	0.9947	0.995454	Genes down-regulated in hepatocellular carcinoma (HCC) induced by diethylnitrosamine (DENA) [PubChem=5921].
LEE_LIVER_CANCER_CIPROFIBRATE_DN	49	-0.11516	-0.474371	0.997	0.995454	Genes down-regulated in hepatocellular carcinoma (HCC) induced by ciprofibrate [PubChem=2763].
